# CXCR4 Signaling Has a CXCL12-Independent Essential Role in Murine *MLL-AF9*-Driven Acute Myeloid Leukemia

**DOI:** 10.1016/j.celrep.2020.107684

**Published:** 2020-05-26

**Authors:** Ramprasad Ramakrishnan, Pablo Peña-Martínez, Puneet Agarwal, Maria Rodriguez-Zabala, Marion Chapellier, Carl Högberg, Mia Eriksson, David Yudovich, Mansi Shah, Mats Ehinger, Björn Nilsson, Jonas Larsson, Anna Hagström-Andersson, Benjamin L. Ebert, Ravi Bhatia, Marcus Järås

**Affiliations:** 1 Division of Clinical Genetics, Lund University, Lund 22184, Sweden; 2 Division of Hematology & Oncology, University of Alabama Birmingham, Birmingham, AL 35233, USA; 3 Division of Molecular Medicine and Gene Therapy, Lund University, Lund 22184, Sweden; 4 Division of Pathology, Department of Clinical Sciences, Skåne University Hospital, Lund University, Lund 22184, Sweden; 5 Division of Hematology and Transfusion Medicine, Lund University, Lund 22184, Sweden; 6 Division of Hematology, Brigham and Women’s Hospital, Boston, MA 02115, USA; 7 Lead Contact

## Abstract

Acute myeloid leukemia (AML) is defined by an accumulation of immature myeloid blasts in the bone marrow. To identify key dependencies of AML stem cells *in vivo*, here we use a CRISPR-Cas9 screen targeting cell surface genes in a syngeneic *MLL-AF9* AML mouse model and show that CXCR4 is a top cell surface regulator of AML cell growth and survival. Deletion of *Cxcr4* in AML cells eradicates leukemia cells *in vivo* without impairing their homing to the bone marrow. In contrast, the CXCR4 ligand CXCL12 is dispensable for leukemia development in recipient mice. Moreover, expression of mutated *Cxcr4* variants reveals that CXCR4 signaling is essential for leukemia cells. Notably, loss of CXCR4 signaling in leukemia cells leads to oxidative stress and differentiation *in vivo*. Taken together, our results identify CXCR4 signaling as essential for AML stem cells by protecting them from differentiation independent of CXCL12 stimulation.

## In Brief

In an *in vivo* CRISPR screen, Ramakrishnan et al. identify CXCR4 as a critical regulator of AML stem cells. Although the CXCR4 ligand CXCL12 is dispensable for leukemia development, CXCR4 signaling is essential for AML cells because it protects them from differentiation.

## Graphical Abstract

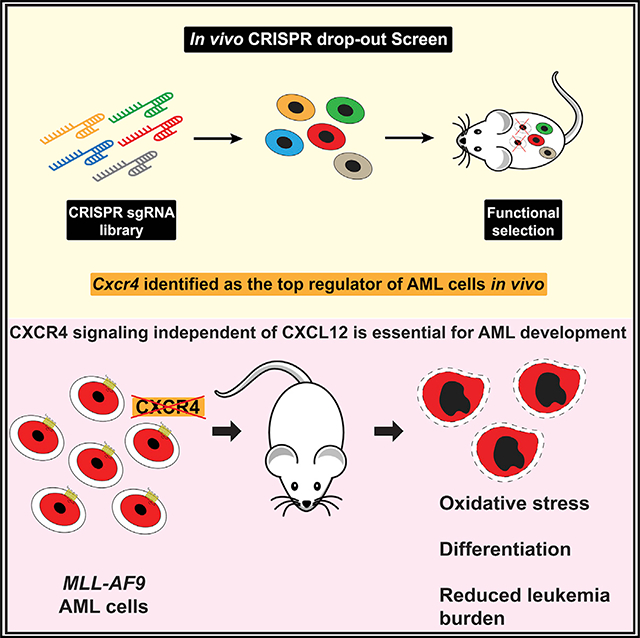

## INTRODUCTION

Acute myeloid leukemia (AML) is a clonal disorder characterized by accumulation of immature, abnormally differentiated myeloid cells in the bone marrow. In adults, AML is the most common acute leukemia and is associated with poor survival (Döhner et al., 2015). The disease is sustained by a small population of leukemic cells, termed leukemia stem cells (LSCs), with self-renewal capacity (Dick, 2005). Within the bone marrow, leukemic cells modulate the microenvironment in a manner that promotes leukemia progression over normal blood cell development and contributes to protection from chemotherapy (Schepers et al., 2015; Shlush et al., 2017; Welner et al., 2015). In addition to anchoring LSCs to the bone marrow niche, binding of ligands to cell surface receptors on LSCs triggers cell signaling, which regulates core components of the LSC entity, such as self-renewal, proliferation, differentiation, and localization (Schepers et al., 2015). Certain cell surface receptors, including CD44, CD123, CD99, CD97, and IL1RAP, which are upregulated on LSCs compared with normal hematopoietic stem and progenitor cells (HSPCs), have been shown to convey signals that support leukemia progression (Charrad et al., 1999; Chung et al., 2017; Järås et al., 2010; Jordan et al., 2000; Martin et al., 2019). Such interactions can also facilitate immune evasion, as exemplified by upregulation of CD47 on leukemia cells that inhibit phagocytes by binding to SIRP-α (Majeti et al., 2009). Many of these cell surface proteins have been successfully explored as therapeutic targets in preclinical models using blocking antibodies and antibodies that direct the immune system to leukemia cells (Ågerstam et al., 2015; Jin et al., 2006, 2009; Tseng et al., 2013). These studies highlight that identifying cell surface proteins upregulated on LSCs relative to normal HSPCs may not only contribute to our understanding of the intricate processes that regulate LSCs, but it can also reveal new therapeutic opportunities. However, because not all upregulated cell surface proteins on LSCs may be functionally important for their growth and survival, there is a need to develop tools that identify cell surface proteins that are biologically important for LSCs under physiological conditions.

A powerful approach to identify genes that are critical for leukemia cells *in vivo* is to perform forward genetic screens. By applying *in vivo* RNA interference (RNAi) screens in a murine AML model, we and others have identified several leukemia-specific dependencies (Järås et al., 2014; Miller et al., 2013; Vu et al., 2017; Zuber et al., 2011). Although powerful, RNAi screens are associated with a high rate of off-target effects and, in recent years, have often been replaced by CRISPR-based methods because these are associated with higher specificity and efficacy (Liberali et al., 2015).

In this study, we performed an *in vivo* pooled CRISPR screen that targeted selected cell surface genes that are upregulated in murine *MLL-AF9* (*KMT2A-MLLT3*) LSC-enriched cells and identified CXCR4 as the top regulator of leukemia-initiating cells. Notably, CXCR4 signaling was found to be essential for LSCs independent of its ligand CXCL12 (SDF-1) *in vivo* by protecting them from differentiation.

## RESULTS

### CRISPR Screening Identifies *In Vivo* Dependencies of *MLL-AF9*-Driven Leukemia

To identify cell surface proteins critical for AML cells *in vivo*, we generated a CRISPR single guide RNA (sgRNA) library targeting 96 cell surface genes (Table S1; Figures S1A–S1C). The genes targeted were selected based on their upregulation in leukemic granulocyte-monocyte progenitor (GMP; Lin^−^Sca-1^−^c-Kit^+^CD34^+^FcγRII/III^+^) cells, enriched for LSCs in the *MLL-AF9*-driven murine leukemia model, relative to normal GMPs, according to global gene expression data (Table S1; Krivtsov et al., 2006; Wang et al., 2010).

**Figure 1.**
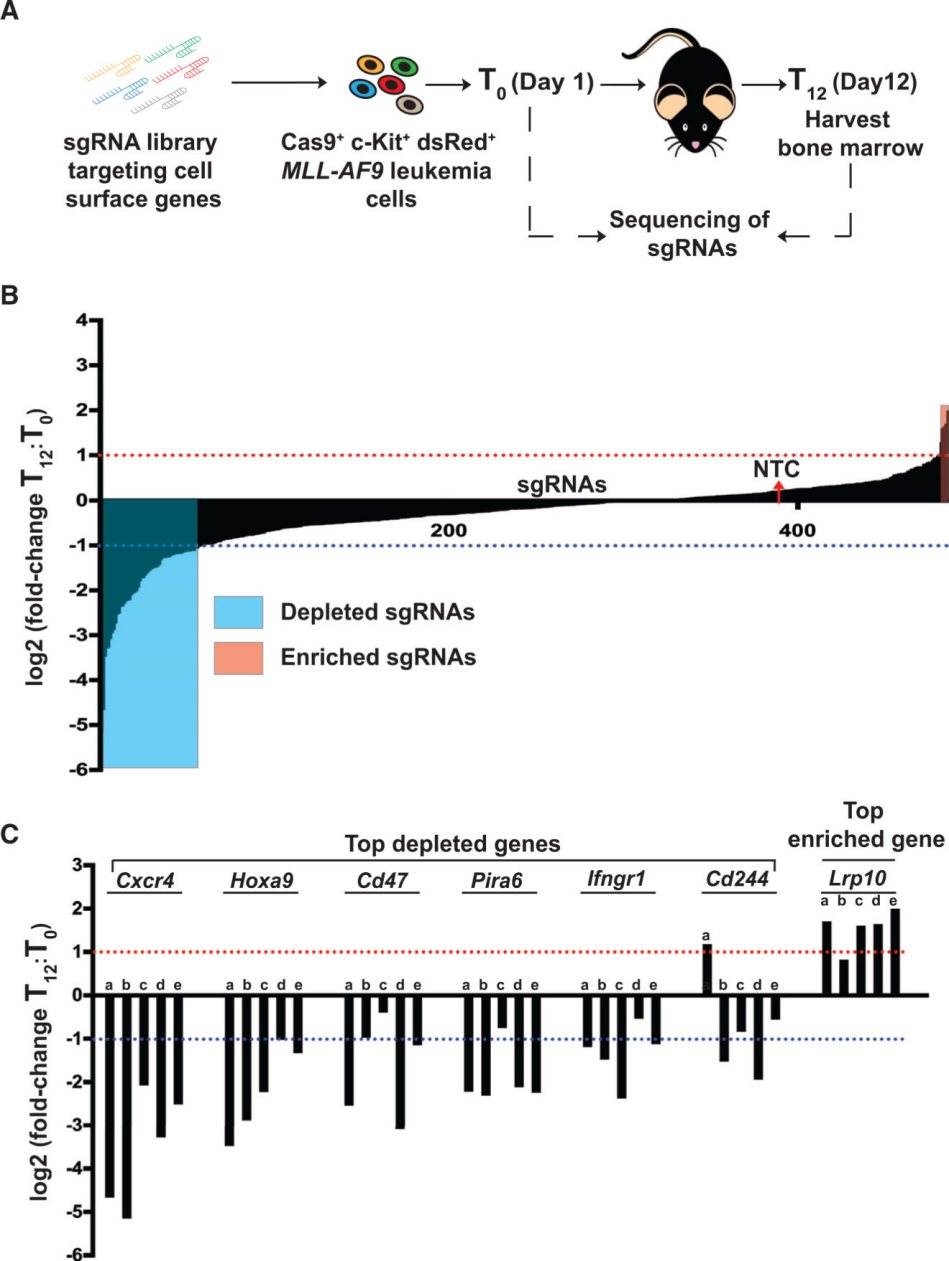
CRISPR Screening Identifies *Cxcr4* as a Critical Regulator of AML Cells *In Vivo* (A) Schematic of the pooled *in vivo* CRISPR screen. In brief, 1 × 10^6^ Cas9^+^dsRed^+^ *MLL-AF9* leukemia cells were transduced with the lentiviral CRISPR sgRNA library targeting selected murine cell surface genes and transplanted into mice (n = 5) after 24 h (T_0_). Twelve days (T_12_) after transplantation, the mice were sacrificed, and the bone marrow was harvested. The representation of sgRNAs was determined by next-generation sequencing (NGS) on genomic DNA of leukemic cells from T_0_ and T_12_. (B) Waterfall plot showing the log2 fold change of the 486 unique sgRNAs at T_12_ versus T_0_ in a ranked format. The red dotted line indicates the threshold for the sgRNAs that were enriched (red box), and the blue dotted line indicates the threshold for depleted (blue box) sgRNAs in the screen. The red arrow denotes the nontargeting control (NTC) sgRNA. (C) Waterfall plot showing the log2 fold change of sgRNAs for the top-ranked genes in the screen. The red dotted line indicates the threshold for the sgRNAs that were enriched, and the blue dotted line indicates the threshold for depleted sgRNAs in the screen. The log2 fold change threshold of 1.0 corresponds to an absolute fold change of 2.0. See also Figure S1 and Tables S1, S2, and S3.

For the CRISPR screen, we used an *MLL-AF9*-driven murine leukemia model we generated previously in a dsRed-transgenic background, allowing convenient tracking of leukemia cells upon serial transplantation in recipient mice (Miller et al., 2013). This AML model is suitable for screens because of its short disease latency and high penetrance (Chapellier et al., 2019; Krivtsov et al., 2006; Puram et al., 2016). Cas9-expressing *MLL-AF9* leukemia cells (Peña-Martínez et al., 2018) were enriched for LSCs by c-Kit selection (Figure S1D), transduced with the sgRNA pool, and transplanted into five sublethally irradiated recipient mice (Figure 1A). To assess the input representation of sgRNAs within the pool, a fraction of the cells was harvested 24 h (T_0_) after transduction. Twelve days after transplantation (T_12_), the bone marrow of recipient mice was harvested, genomic DNA was extracted, and the sgRNAs were PCR amplified prior to sequencing. Based on the number of sgRNAs per gene that were either enriched or depleted beyond a threshold of a median fold change of 2.0 across 5 biological replicates, a ranked gene list of candidate LSC regulators was generated (Figures 1B and 1C; Table S2). Although representation of a nontargeting control sgRNA did not change significantly *in vivo*, 5 of 5 sgRNAs targeting the positive control *Hoxa9*, essential for *MLL-AF9* leukemia cells (Faber et al., 2009), showed a median depletion of more than 2-fold, demonstrating that the screen was robust (Figure 1C). The genes that ranked as the most important positive regulators for LSCs were *Cxcr4*, *Cd47*, *Pira6*, *Ifngr1*, and *Cd244*; at least two sgRNAs targeting these genes showed a depletion of a fold change of more than 2.0 (Figure 1C). *Lrp10* was identified as the only negative regulator of LSCs, with 4 of 5 sgRNAs showing more than 2-fold enrichment. By generating lentiviral vectors expressing sgRNAs targeting *Lrp10* from the screen and with coexpression of tRFP657, the findings from the screen could be successfully validated, with a significant enrichment of sgRNAs *in vivo* upon leukemia development in mice (p < 0.01) (Figure S1E).

### CXCR4 Is Critical for *MLL-AF9* Leukemia Cell Growth and Survival *In Vivo*

*Cxcr4* was identified as the top-ranked positive regulator of leukemia cells, with all 5 sgRNAs showing a median depletion of a fold change of more than 2.0 *in vivo* and with one sgRNA depleted more than 30-fold (5.1-fold depletion in log2 scale) (Figure 1C). High expression of CXCR4 in AML cells has been associated previously with poor prognosis (Spoo et al., 2007; Ahn et al., 2013; Du et al., 2019). In transcriptomics data from AML patients (Ley et al., 2013), we found mildly but significantly increased expression of *Cxcr4* in the M5 (monocytic differentiation) subtype and in *MLL*-rearranged AML (Figures S2A and S2B). Although CXCR4 has been shown to regulate leukemia-initiating cells in T cell acute lymphoblastic leukemia (Passaro et al., 2015), the functional role of CXCR4 in AML development has remained elusive (Monaco et al., 2004; Tavor et al., 2004) and has so far not been investigated using genetic deletion of *Cxcr4* in AML cells. Thus, to unravel how CXCR4 regulates the growth and survival of *MLL-AF9* AML cells under syngeneic conditions, we selected CXCR4 for follow-up studies.

**Figure 2.**
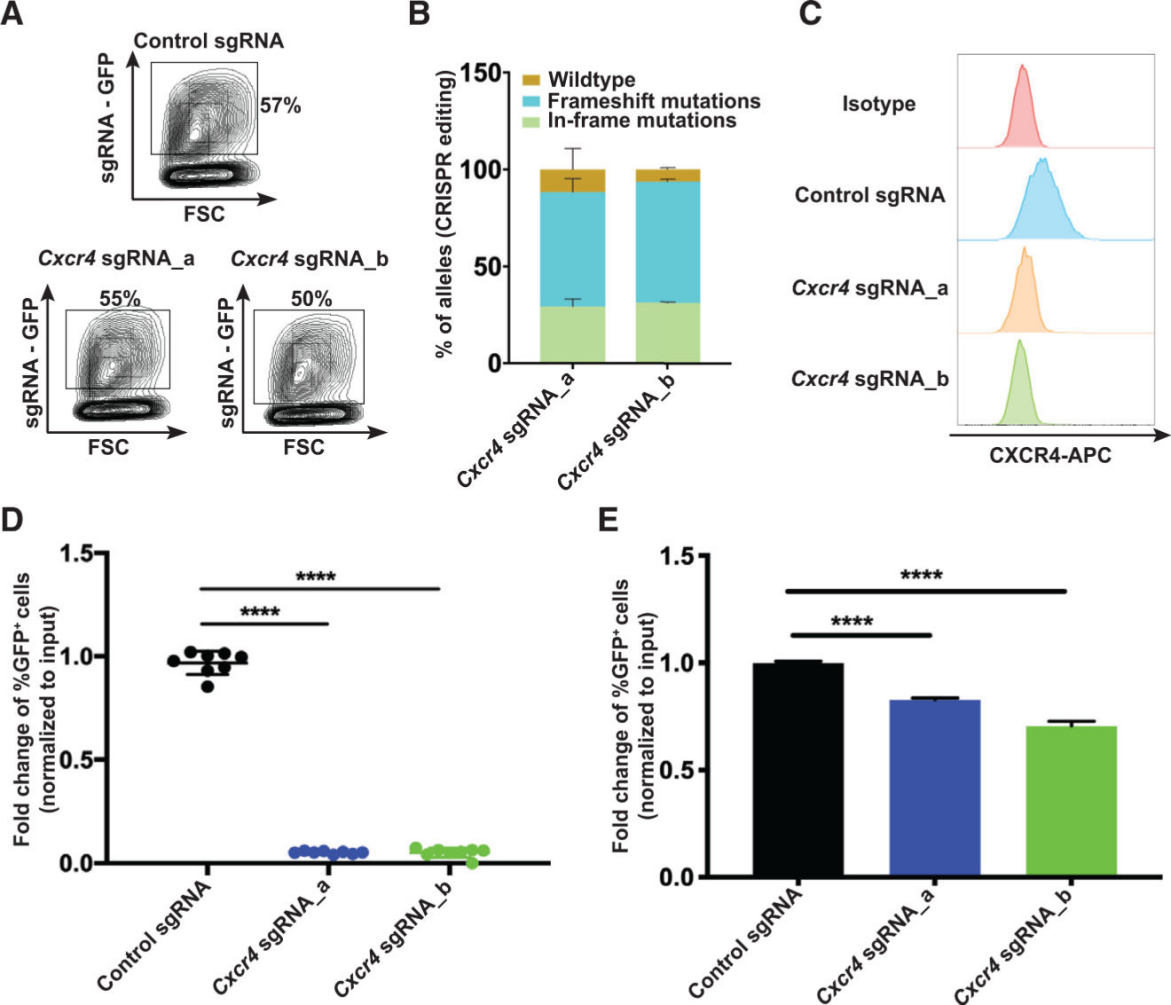
CXCR4 Is Critical for Growth and Survival of Leukemia Cells *In Vivo* (A) Green fluorescent protein (GFP) expression inCas9^+^dsRed^+^ leukemia cells following transduction with lentiviral vectors coexpressing control or *Cxcr4* sgRNAs and GFP. (B) Quantification of CRISPR editing in *Cxcr4* by NGS within sorted GFP^+^ leukemia cells 3 days after transduction. (C) CXCR4 cell surface expression as measured by flow cytometry on dsRed^+^ leukemia cells expressing sgRNAs targeting *Cxcr4* 3 days after transduction. (D) Mice (n = 8 for each group) were transplanted with leukemia cells transduced with lentiviral vectors coexpressing *Cxcr4* sgRNAs and GFP. The mice were sacrificed 12 or 13 days after transplantation. The percentage of GFP^+^ cells in the bone marrow was normalized to the input percentage of GFP^+^ cells 2 days after transduction to compensate for the differences in transduction efficiency between groups. (E) Leukemia cells were transduced with lentiviral vectors coexpressing *Cxcr4* sgRNAs and GFP, and the transduction efficiency was determined after 3 days (input). The cells were cultured without cytokine stimulation for 3 more days (n = 3), and the percentage of GFP^+^ cells was normalized to the input. Means and standard deviations are shown (****p < 0.0001). See also Figures S2 and S3.

**Figure 3.**
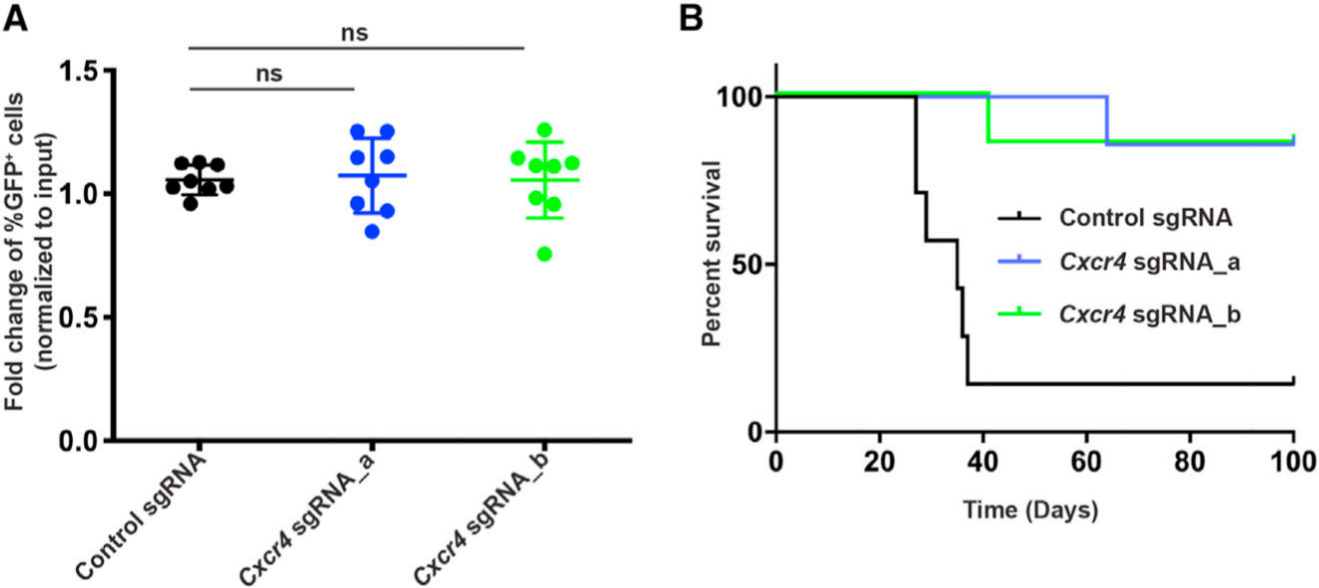
CXCR4 Is Essential for AML Cells *In Vivo* but Not for Homing of Leukemic Cells to the Bone Marrow (A) Percentage of GFP^+^ leukemia cells among dsRed^+^ cells in the bone marrow 24 h after transplantation (n = 8 for each group). The data were normalized to the percentage of GFP^+^ cells for each sample prior to injection 3 days after transduction (input). (B) Survival of recipient mice (n = 7 for each group) transplanted with 40,000 sorted GFP^+^ leukemia cells 3 days after transduction with sgRNA-expressing vectors. ns, not significant. See also Figure S4.

We first validated the importance of *Cxcr4* in AML by transducing Cas9^+^dsRed^+^ *MLL-AF9* leukemia cells with two different sgRNA-expressing vectors targeting *Cxcr4* and coexpressing GFP (Figure 2A). Both sgRNAs induced more than 90% gene editing in the *Cxcr4* locus, resulting in loss of CXCR4 expression (Figures 2B and 2C). We next studied how *Cxcr4* disruption affects leukemia cells under physiological conditions by performing a competition assay between GFP^+^ (sgRNA-expressing) and GFP^−^ cells *in vivo*. Consistent with our findings from the screen, GFP^+^ leukemia cells expressing *Cxcr4* sgRNAs transplanted into sublethally irradiated recipient mice showed strong depletion in the bone marrow and spleen compared with GFP^+^ leukemia cells expressing a control sgRNA (Figure 2D; Figure S3A). In contrast, a corresponding competition assay *in vitro* with standard cytokines (interleukin-3 [IL-3], IL-6, and stem cell factor [SCF]) demonstrated that disruption of *Cxcr4* in c-Kit^+^ leukemia cells did not affect cell proliferation (FigureS3B). However, by culturing leukemia cells under low stimulatory conditions without cytokines, mild but significant depletion of *Cxcr4* sgRNA-expressing GFP^+^ leukemia cells was observed, suggesting that CXCR4 provides signaling that supportscellular growth and survival (Figure 2E). Combined, these data reveal a critical role of CXCR4 in AML cell growth and survival *in vivo*.

### CXCR4 Is Not Critical for Homing of Leukemia Cells to the Bone Marrow but Essential for AML Development

In normal hematopoiesis, CXCR4 is critical for homing and retention of hematopoietic stem cells (HSCs) in the bone marrow (Foudi et al., 2006; Sugiyama et al., 2006); however, it is unclear whether CXCR4 is involved in homing of AML cells to the bone marrow. Although Tavor et al. (2004) found that homing of primary human AML cells to the bone marrow of non-obese diabetic (NOD)/severe combined immunodeficiency (SCID)/B2m^null^ mice is CXCR4 dependent, Monaco et al. (2004) reported that CXCR4 is dispensable for repopulation of human AML cells in NOD/SCID mice. To clarify the role of CXCR4 in homing of AML cells in a syngeneic AML model, Cas9^+^ *MLL-AF9* leukemia cells were transduced with the *Cxcr4* or control sgRNA vectors, and 3 days later, cells were transplanted into sublethally irradiated mice. The percentage of GFP^+^ cells in the bone marrow was assessed 24 h after transplantation. No significant differences in the percentages of GFP^+^ cells were observed at this time point within the leukemic graft (Figure 3A; Figure S4A). Consistent with this, when GFP^+^ (sgRNA-expressing) leukemia cells were sorted prior to the homing assay, there was no significant difference in the percentage of GFP^+^ cells in the bone marrow 24 h after transplantation (Figure S4B), suggesting that CXCR4 is not critical for *MLL-AF9* leukemia cell homing to the bone marrow.

To further assess whether CXCR4 is essential for full leukemia development *in vivo*, we sorted GFP^+^ (sgRNA-expressing) leukemia cells and transplanted them into sublethally irradiated mice. Although the majority of the mice in the control group developed leukemia, only two mice transplanted with leukemia cells expressing *Cxcr4* sgRNAs developed leukemia (Figure 3B; Figures S4C and S4D). Interestingly, the leukemia cells in these two mice expressed normal CXCR4 levels in the bone marrow and spleen, suggesting that the cells had escaped silencing (Figure S4E). These findings demonstrate that CXCR4 is not required for homing of AML cells to the bone marrow but essential for leukemia development *in vivo*.

### Loss of CXCR4 Results in Oxidative Stress and Differentiation of Leukemia Cells

**Figure 4.**
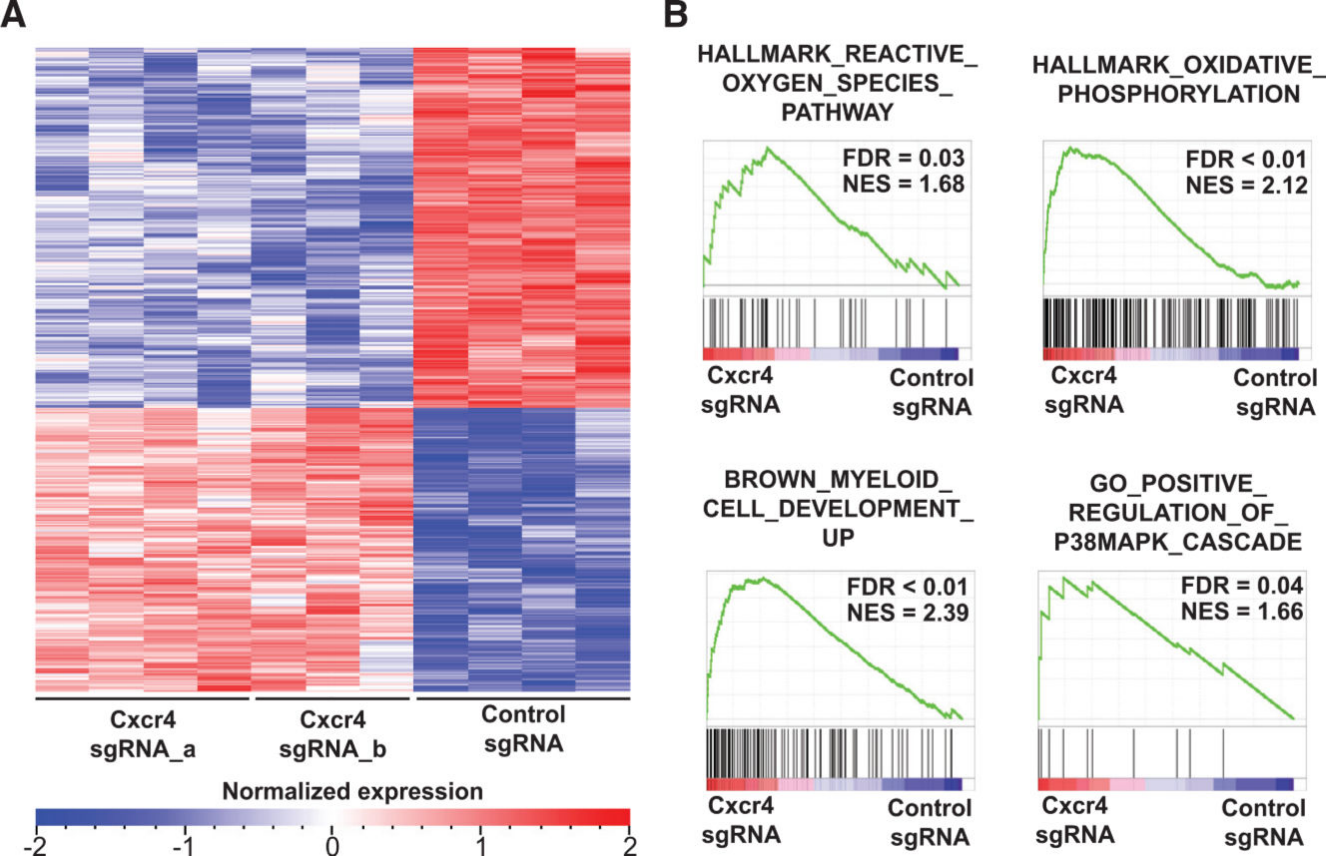
CXCR4 Disruption in Leukemia Cells Results in a Distinct Gene Expression Signature Associated with Oxidative Stress and Differentiation (A) Heatmap of the differentially expressed genes(473 genes, false discovery rate [FDR] < 0.01) from RNA sequencing performed on sorted GFP^+^ (sgRNA-expressing) cells harvested from the bone marrow of recipient mice (n = 4 for control sgRNA and Cxcr4 sgRNA_a; n = 3 for Cxcr4 sgRNA_b) transplanted with *MLL-AF9* leukemia cells. (B) Gene set enrichment analysis (GSEA) of the transcriptional signature obtained upon *Cxcr4* disruption. NES, normalized enrichment score. See also Figure S5.

**Figure 5.**
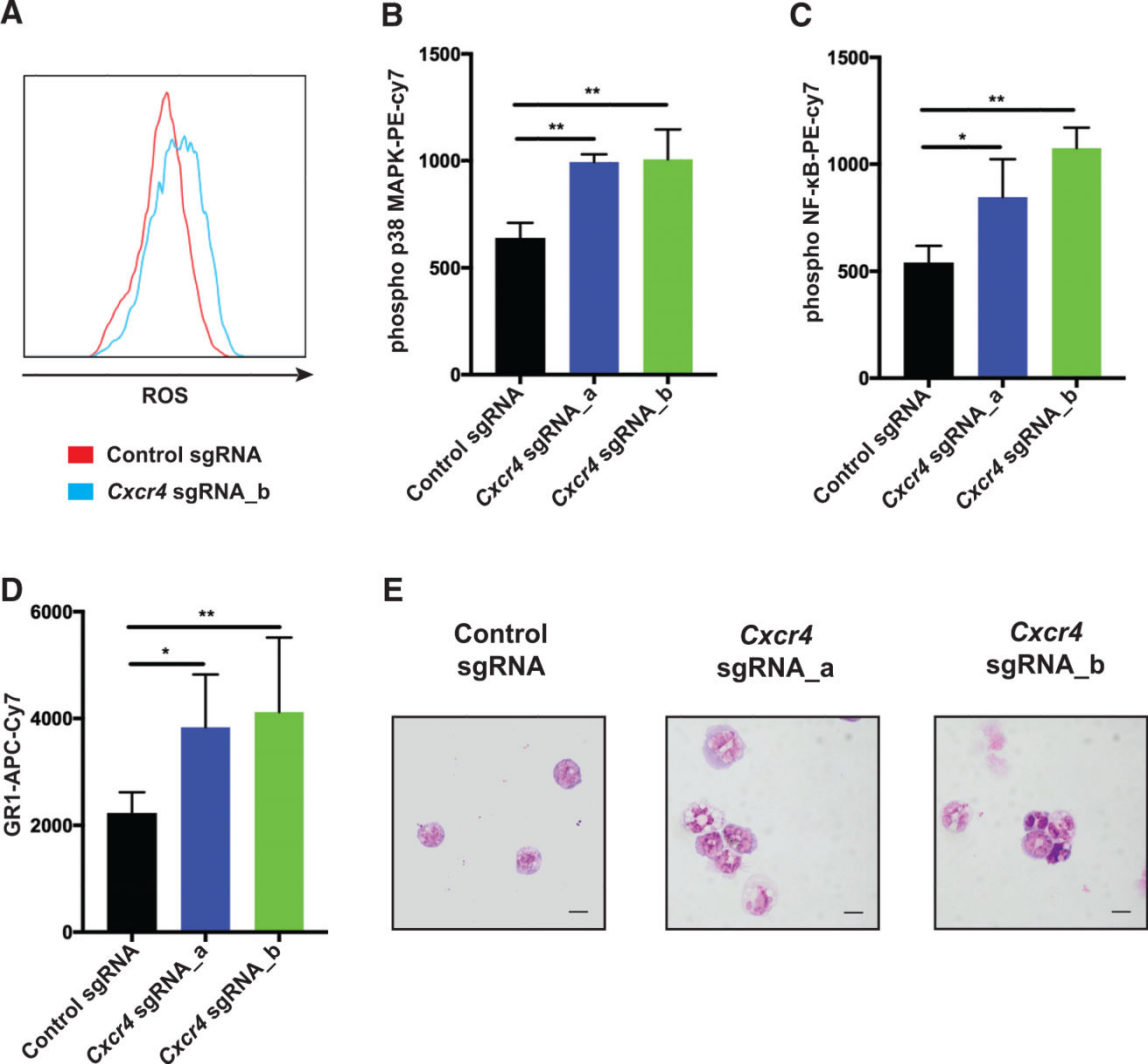
Loss of CXCR4 Leads to Differentiation of Leukemia Cells (A) Representative histogram showing total ROS levels (measured with CellROX Deep Red) in c-Kit^+^ cells within GFP^+^ (sgRNA-expressing) cells harvested from the bone marrow of recipient mice (n = 5 for control sgRNA; n = 4 for *Cxcr4* sgRNA_b) 14 days after transplantation. (B–D) Mean fluorescence intensity (MFI) of (B) phosphorylated p38 MAPK, (C) phosphorylated NF-κB, and (D) Gr-1 within GFP^+^ (sgRNA-expressing) leukemia cells that were harvested from the bone marrow of recipient mice (n = 3 for each group) 10 days after transplantation. (E) May-Grünwald-Giemsa-stained cytospin slides from representative sgRNA-expressing leukemia cells 13 days after transplantation. Scale bars represent 10 μm. Means and standard deviations are shown (*p < 0.05, **p < 0.01, ***p < 0.001). See also Figure S5.

To assess how *Cxcr4* deletion in c-Kit^+^ *MLL-AF9* leukemia cells affects the fate of leukemia cells *in vivo*, we next performed RNA sequencing of sorted *Cxcr4* sgRNA-expressing leukemia cells harvested from mice 10 days after transplantation. *Cxcr4* disruption resulted in a distinct gene expression signature (Figure 4A). By gene set enrichment analysis (GSEA), we found that the signature was enriched for gene sets related to reactive oxygen species (ROS) pathways and oxidative phosphorylation (Figure 4B). In normal HSCs, disruption of *Cxcr4* in mice has been shown to result in oxidative stress, leading to activation of p38 mitogen-activated protein kinase (MAPK) and loss of HSCs (Ito et al., 2006; Zhang et al., 2016). In line with this, gene sets for p38 MAPK signaling and myeloid differentiation were enriched in the *Cxcr4*-disruption signature in leukemia cells (Figure 4B). In addition, GSEA also revealed enrichment of genes associated with nuclear factor κB (NF-κB) signaling (Figure S5A). Consistent with the transcriptome analysis, in the *Cxcr4*-disrupted leukemia cells, we observed a significant increase in ROS in both the LSCs (c-Kit^+^) and the bulk leukemia cells *in vivo* (Figure 5A; Figure S5B–S5D). In addition, there was enhanced phosphorylation of p38 MAPK and NF-κB and upregulation of the myeloid differentiation marker Gr-1 (Figures 5B–5D; Figures S5E and S5F). Morphological assessments of these cells identified changes associated with granulocytic differentiation (Figure 5E). Taken together, these findings suggest that loss of CXCR4 in leukemia cells leads to increased oxidative stress, differentiation, and activation of p38 and NF-κB signaling.

### CXCL12 Expression in the Microenvironment Is Dispensable for AML Development

**Figure 6.**
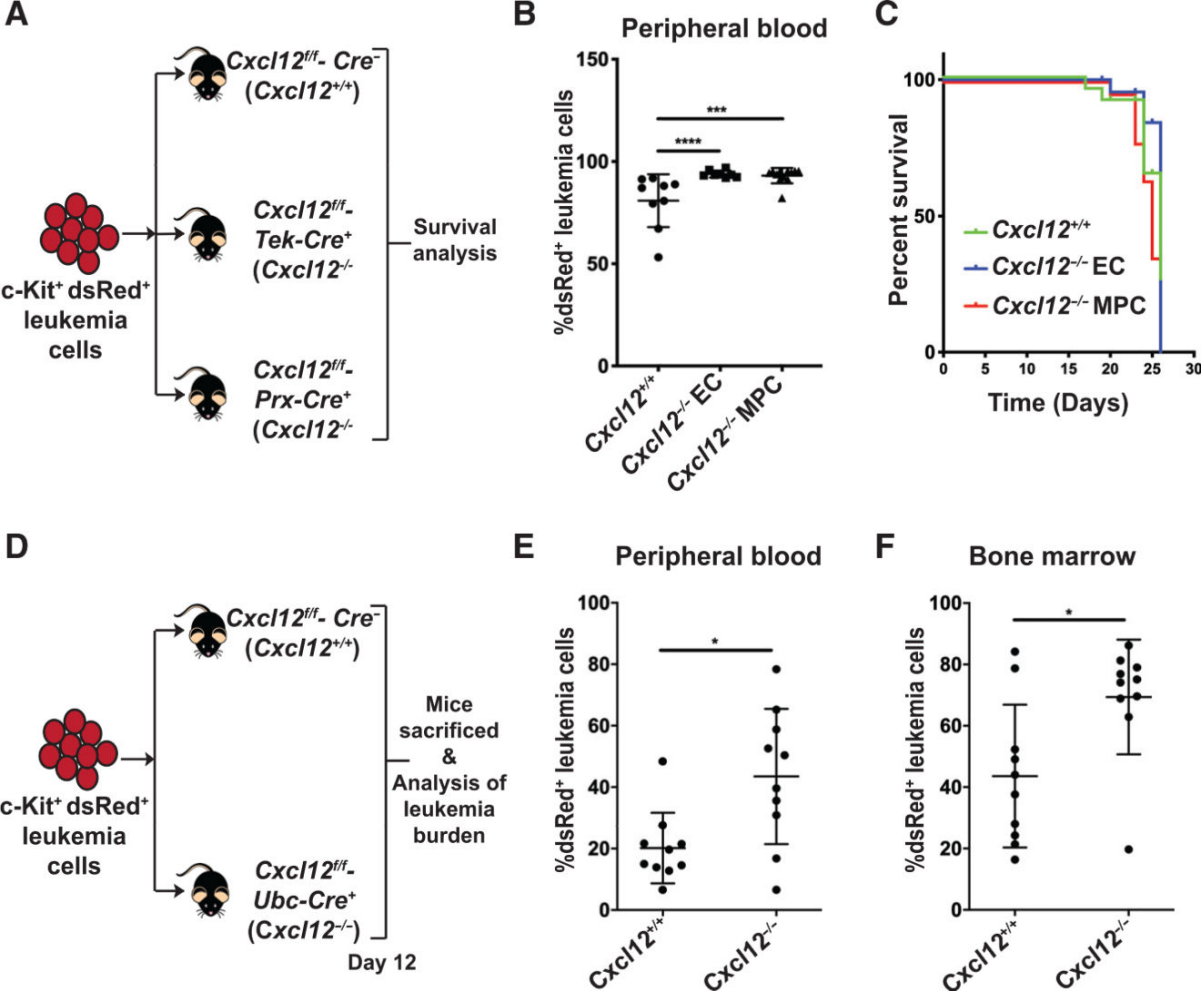
CXCL12 Expression in the Bone Marrow Restrains AML Development (A) Schematic of transplantations of c-Kit^+^dsRed^+^ leukemia cells into *Cxcl12*^+/+^ recipient mice (n = 9), *Cxcl12*^*f/f*^-*Tek-Cre*^+^ (*Cxcl12*^−/−^ ECs) recipient mice (n = 8), and *Cxcl12*^*f/f*^-*Prx1-Cre*^+^ (*Cxcl12*^−/−^ MPCs) recipient mice (n = 12), followed by survival analysis. (B and C) Percentage of dsRed^+^ leukemia cells in the peripheral blood of recipient mice 16 days after transplantation (B) and subsequent survival analysis (C). (D) Schematic of transplantations of c-Kit^+^dsRed^+^ leukemia cells into *Cxcl12*^+/+^ (n = 10) and *Cxcl12*^*f/f*^-*Ubc-Cre*^+^ (*Cxcl12*^−/−^) recipient mice (n = 10) and subsequent analysis of the leukemia burden. (E and F) Percentage of dsRed^+^ leukemia cells 12 days after transplantation in (E) peripheral blood and (F) bone marrow. Means and standard deviations are shown (*p < 0.05, ***p < 0.001, ****p < 0.0001). See also Figure S6.

CXCL12 is the main ligand for CXCR4 and a homeostatic chemokine widely expressed by several cell types in the bone marrow, existing as a membrane-bound protein and in soluble form (Nagasawa, 2014). In culture, binding of CXCL12 to CXCR4 has been shown to promote leukemia cell proliferation and trans-well migration (Liesveld et al., 2007; Möhle et al., 1998; Tavor et al., 2004). Consistent with these studies, high CXCL12 levels stimulated survival of *MLL-AF9* AML cells in culture (Figure S6A). Under physiological conditions, CXCL12 expression in the bone marrow microenvironment is necessary for keeping HSCs in a quiescent state (Tzeng et al., 2011). In particular, CXCL12 expression in endothelial cells and mesenchymal progenitor cells is needed for retention of normal HSPCs in the bone marrow (Ding and Morrison, 2013; Greenbaum et al., 2013). However, it is unknown whether CXCL12 is also critical for regulating AML cells. To assess whether CXCL12 expression by endothelial cells (ECs) or mesenchymal progenitor cells (MPCs) regulates AML cells, we transplanted c-Kit^+^ *MLL-AF9* cells into *Cxcl12*^f/f^-*Tek*-Cre^+^ (*Cxcl12*^−/−^ ECs) and *Cxcl12*^f/f^-*Prx1*-Cre^+^ (*Cxcl12*^−/−^ MPCs) recipient mice with *Cxcl12* knocked out in ECs and MPCs, respectively (Figure 6A). In the peripheral blood, 16 days after transplantation, we found a mild increase in leukemia cells in *Cxcl12*^−/−^ EC mice and *Cxcl12*^−/−^ MPC mice compared with *Cxcl12*^+/+^ recipient mice (Figure 6B), but no difference in survival between the groups was observed (Figure 6C). Thus, loss of *Cxcl12* expression in ECs and MPCs is dispensable for leukemia development. Upon sacrifice, all mice had fully developed leukemia in the bone marrow and spleen (Figures S6B and S6C). To further assess whether CXCL12, provided by the microenvironment, affects the growth and survival of leukemia cells, we transplanted c-Kit^+^ *MLL-AF9* leukemia cells into *Cxcl12*^f/f^-*Ubc*-Cre^+^ (referred to as *Cxcl12*^−/−^) mice (Figure 6D; Figure S6D), which are devoid of CXCL12 expression in all tissues. Relative to *Cxcl12*^+/+^ control mice, we observed an increase in leukemia cell levels in *Cxcl12*^−/−^ recipient mice in peripheral blood and bone marrow (Figures 6E and 6F). No significant difference in cell cycle status and CXCR4 expression was observed for the leukemia cells between *Cxcl12*^−/−^ and *Cxcl12*^+/+^ recipient mice (Figures S6E and S6F). These data suggest that, in contrast to what has been reported for normal HSPCs, the CXCR4-CXCL12 interaction has a mild negative effect on *MLL-AF9* AML development in mice.

### CXCR4 Signaling Independent of CXCL12 Is Essential for AML Development

**Figure 7.**
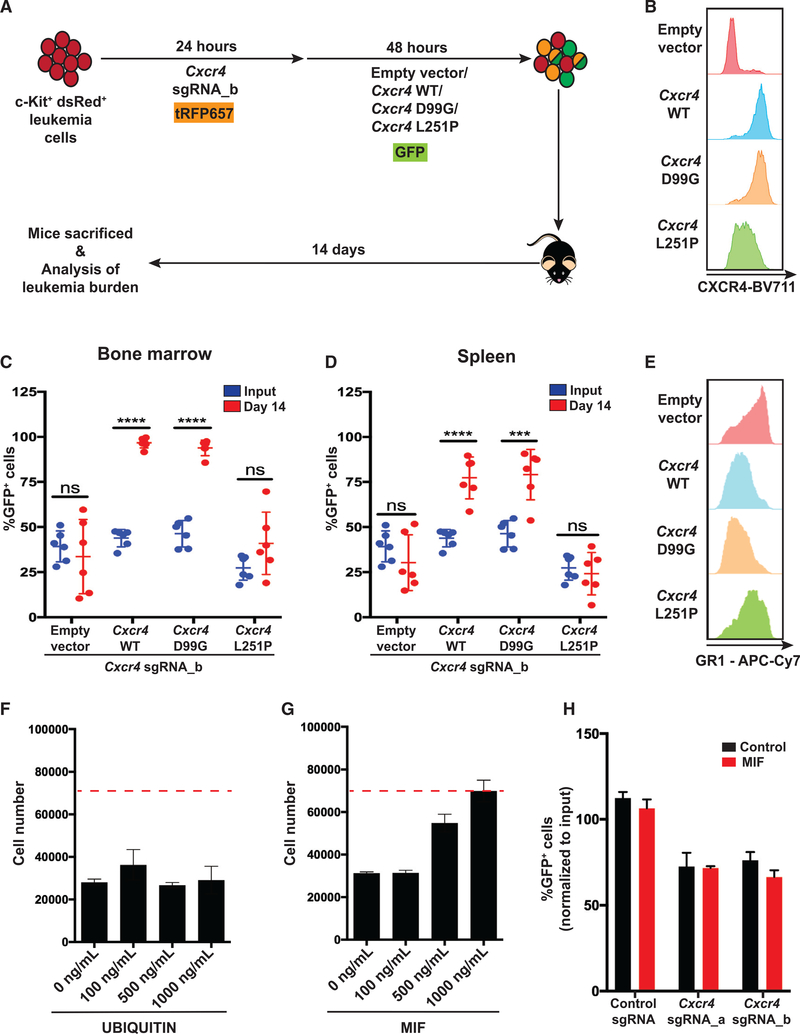
CXCR4 Signaling Independent of CXCL12 Stimulation Is Essential for AML Cells *In Vivo* (A) Schematic of the *in vivo* rescue assay with *Cxcr4* mutants. Cas9^+^c-Kit^+^ dsRed leukemia cells were sequentially transduced with the *Cxcr4* sgRNA_b lentiviral vector coexpressing tRFP657, followed by transduction with a retroviral control vector, *Cxcr4*^*WT*^, or a CXCL12-resistant *Cxcr4* mutant (*Cxcr4*^*L251P*^ or *Cxcr4*^*D99G*^)-expressing vector and transplanted into sublethally irradiated mice. (B) Representative histograms showing CXCR4 expression in the GFP^+^ population among dsRed^+^tRFP657^+^ leukemia cells 3 days after transduction. (C and D) Percentage of GFP^+^ cells within the dsRed^+^tRFP657^+^ leukemia cells in the input cells and in the (C) bone marrow and (D) spleen of recipient mice (n = 6 for each group) 14 days after transplantation. (E) Representative histograms showing Gr-1 expression 11 days after transplantation within GFP^+^dsRed^+^tRFP657^+^ leukemia cells extracted from the bone marrow of mice (n = 3 for each group) transplanted with leukemia cells transduced with different *Cxcr4* variants. (F and G) Cell number after 3 days of culture of 70,000 seeded dsRed^+^ leukemia cells (red dotted line) exposed to increasing concentrations of (F) UBIQUITIN (n = 3) and (G) MIF (n = 3). (H) Percentage of GFP^+^ leukemia cells normalized to the input (3 days after transduction) when cultured *in vitro* for 3 days in serum-free medium without any cytokines (control) or with 1 μg/mL MIF (n = 3). Means and standard deviations are shown (***p < 0.001, ****p < 0.0001). See also Figure S7.

To further characterize how CXCR4 signaling promotes leukemia development, we generated two retroviral vectors expressing murine *Cxcr4* mutant cDNAs: *Cxcr4*^*D99G*^ and *Cxcr4*^*L251P*^. CXCR4^D99G^ (corresponding to human CXCR4^D97G^) has an amino acid substitution in the extracellular domain of the receptor and lacks the ability to bind to CXCL12 (Choi et al., 2005; Wescott et al., 2016), and CXCR4^L251P^ (corresponding to human CXCR4^L244P^) has an amino acid substitution in a transmembrane signaling domain, resulting in a signaling-dead receptor (Wescott et al., 2016; Figure S7A). We also generated a retroviral vector expressing a wild-type *Cxcr4* cDNA used as a reference (*Cxcr4*^*WT*^). In all CXCR4 cDNAs, we inserted silent mutations to make them resistant to *Cxcr4* sgRNAs. We sequentially transduced *MLL-AF9* leukemia cells with *Cxcr4* sgRNA_b coexpressing tRFP657 and *Cxcr4* cDNAs coexpressing GFP, which resulted in restored CXCR4 expression on the cell surface (Figures 7A and 7B; Figure S7B). As anticipated, leukemia cells expressing *Cxcr4*^*D99G*^ or *Cxcr4*^*L251P*^ did not respond to CXCL12 stimulation in culture, whereas *Cxcr4*^*WT*^-expressing cells responded (Figure S7C). We also confirmed that *Cxcr4*^*L251P*^ is signaling dead by measuring phosphorylation of extracellular-signal-regulated kinase (ERK) following CXCL12 stimulation of leukemia cells (Figure S7D). Next we assessed whether the mutated *Cxcr4* cDNA could rescue depletion of leukemia cells *in vivo* caused by CRISPR-mediated disruption of endogenous *Cxcr4*. Expression of *Cxcr4*^*WT*^ or *Cxcr4*^*D99G*^ rescued depletion of leukemia cells in the bone marrow and spleen (Figures 7C and 7D). Given that CXCR4^D99G^ lacks the ability to bind to CXCL12, the results confirm that CXCL12 is dispensable for the growth and survival of *MLL-AF9* leukemia cells. Importantly, these findings also show that depletion of leukemia cells following *Cxcr4* disruption is on target and not caused by off-target effects of the *Cxcr4* sgRNA. Interestingly, expression of the signaling-dead variant of CXCR4, CXCR4^L251P^, failed to rescue depletion of leukemia cells in the bone marrow and spleen, demonstrating that CXCR4 signaling is essential for leukemia cells (Figures 7C and 7D). Moreover, increased Gr-1 expression following disruption of *Cxcr4* in leukemia cells *in vivo* was abolished by expression of *Cxcr4*^*WT*^ or *Cxcr4*^*D99G*^ but not by the signaling-dead variant, *Cxcr4*^*L251P*^ (Figure 7E). Thus, CXCR4 signaling is essential for AML cells *in vivo* independent of CXCL12 stimulation. In addition, consistent with the increased phosphorylation of NF-κB upon *Cxcr4* disruption (Figure 5C), overexpression of *Cxcr4*^*WT*^ in *Cxcr4*-disrupted leukemia cells resulted in reduced phosphorylation of NF-κB (Figure S7E).

Apart from binding to CXCL12, CXCR4 has been described as one of the receptors for extracellular UBIQUITIN and macrophage migration inhibitory factor (MIF) (Bernhagen et al., 2007; Saini et al., 2010). Because we found that CXCL12 is dispensable for AML development, we next assessed whether UBIQUITIN or MIF promotes growth and survival of *MLL-AF9* leukemia cells by binding to CXCR4. Although UBIQUITIN did not stimulate growth or survival of *MLL-AF9* leukemia cells *in vitro*, there was a slight but dose-dependent increase in leukemia cell numbers when supplementing the culture medium with MIF (Figures 7F and 7G). To address whether MIF promotes leukemia cells in a CXCR4-dependent manner, we evaluated whether leukemia cells with *Cxcr4* disruption responded to MIF. Compared with leukemia cells expressing normal *Cxcr4* levels, *Cxcr4* deletion did not affect MIF-induced growth or survival of leukemia cells (Figure 7H).

Taken together, our data suggest that CXCR4 signaling is essential for growth and survival of *MLL-AF9* leukemia cells independent of CXCL12, MIF, and UBIQUITIN stimulation.

## DISCUSSION

CRISPR screening is a powerful method to identify cancer cell dependencies (Behan et al., 2019; Chan et al., 2019) but is often limited by culture conditions that poorly reflect the *in vivo* tumor microenvironment. We generated a CRISPR library targeting 96 cell surface genes upregulated in LSCs and used it to identify positive and negative regulators of AML cell growth and survival in the bone marrow microenvironment.

*Cxcr4* was identified as the top positive regulator of *MLL-AF9* leukemia cells, with all five *Cxcr4* sgRNAs being depleted *in vivo*. The finding that *Cxcr4* disruption selectively affects growth and survival of leukemia cells *in vivo* demonstrates the limitation of *in vitro* assays to address physiologically relevant dependencies of leukemia cells. It also highlights the importance of performing CRISPR screens in systems where essential interactions between tumor cells and the microenvironment can be detected. CXCR4 has been shown to play a key role in normal HSCs in the bone marrow niche (Nie et al., 2008; Sugiyama et al., 2006; Zou et al., 1998) and has been explored as a therapeutic target in leukemia (Abraham et al., 2017; Liu et al., 2017; Tavor et al., 2008). However, the functional *in vivo* role of CXCR4 in AML has remained elusive; in particular, its role in homing of leukemia cells to the bone marrow, cell signaling, and interactions with CXCL12 is unclear (Monaco et al., 2004; Tavor et al., 2004). Here we used CRISPR-mediated disruption of *Cxcr4* in leukemia cells along with expression of mutated CXCR4 variants and show that, although CXCR4 is dispensable for homing of leukemia cells to the bone marrow, CXCR4 signaling is essential for leukemia development, suggesting that CXCR4 is among the core regulators required for LSC maintenance. In contrast, *Cxcr4*^−/−^ normal HSCs are capable of sustaining long-term hematopoiesis (Nie et al., 2008). Although CXCR4 expression was essential for serial propagation of *MLL-AF9* leukemia cells in mice, it is unclear whether CXCR4 is critical for initiation of *MLL-AF9* leukemia in a primary recipient mouse.

The dramatic loss of AML cells *in vivo* following *Cxcr4* deletion was associated with oxidative stress and granulocytic differentiation, as shown by the morphology, immunophenotype, and activation of the p38 MAPK and NF-κB pathways. Although enhanced NF-κB signaling is associated with myeloid differentiation in normal hematopoiesis and in *MLL-AF9* LSCs (Bottero et al., 2006; Eriksson et al., 2017), the NF-κB subunit, v-rel avian reticuloendotheliosis viral oncogene homolog A (RELA) has also been found to sustain an *MLL*-dependent LSC program (Kuo et al., 2013) and accelerate leukemia development (Xiu et al., 2018). Our data suggest that loss of CXCR4 expression leads to development of leukemia cells that have elevated NF-κB signaling, probably reflecting a more differentiated state of the cells rather than NF-κB signaling driving the differentiation effect itself. An increase in ROS levels has been associated previously with differentiation of normal HSCs (Itkin et al., 2016), and the reduction of HSCs in *Cxcr4*^−/−^ mice is coupled to oxidative stress, DNA damage, and activation of p38 MAPK (Zhang et al., 2016). The reason why LSCs are more sensitive to oxidative stress than normal HSPCs has been linked previously to a lower reserve spare capacity in their respiratory chain complexes (Testa et al., 2016). In line with these findings, an increase in ROS has been shown to be detrimental to *MLL-AF9* AML cells (Roychoudhury et al., 2015). Although we found that *Cxcr4* disruption results in increased ROS and differentiation of leukemia cells, it is currently unclear whether it is elevated ROS levels that cause differentiation of the cells.

Given that the CXCR4-CXCL12 interaction is important for retention and regulation of normal HSPCs in the bone marrow (Ding and Morrison, 2013; Greenbaum et al., 2013), we also studied the role of CXCL12 in AML development. Notably, we found that *MLL-AF9* leukemia was accelerated in lineage-restricted and global *Cxcl12*^−/−^ mice, suggesting that CXCL12 expressed by cells in the bone marrow microenvironment restrains AML development. These findings are partially consistent with recent observations in a transgenic chronic myeloid leukemia (CML) mouse model in which deletion of *Cxcl12* in MPCs promotes expansion of LSCs (Agarwal et al., 2019). However, in contrast to our findings, deletion of *Cxcl12* in ECs in the CML model resulted in depletion of LSCs. This suggest that LSCs in chronic-phase CML are dependent on vascular niches, similar to normal HSPCs (Ding and Morrison, 2013; Greenbaum et al., 2013), whereas in AML, leukemia cells are less dependent on these niches for disease development. Although we did not observe a difference in the cycle status of leukemia cells upon disease development in *Cxcl12*^−/−^ mice, given that CXCL12 keeps normal HSCs in a quiescent state (Tzeng et al., 2011), we speculate that CXCL12 also restrains cell cycle progression of AML cells at the early stages of AML development. Alternatively, deletion of *Cxcl12*, which mobilizes normal HSCs to the peripheral blood (Greenbaum et al., 2013), provides niches in the bone marrow that facilitate expansion of AML cells.

By expressing two CXCR4 mutated variants in AML cells with disrupted endogenous *Cxcr4*, we could show that CXCR4 signaling, but not CXCL12 binding, is essential for *MLL-AF9* leukemia cells *in vivo*. Because deletion of *Cxcr4* resulted in depletion of *MLL-AF9* leukemia cells in serum-free medium without cytokines and because the CXCR4 ligands UBIQUITIN and MIF failed to promote growth and survival of *MLL-AF9* leukemia cells by binding to CXCR4, our data suggest that CXCR4 provides baseline signaling independent of ligand stimulation that is sufficient to promote growth and survival of leukemia cells *in vivo*. This could explain why CXCL12 is dispensable for AML development *in vivo*, whereas *in vitro*, supra-physiological concentrations of CXCL12 promoted survival of leukemia cells in the absence of other cytokine stimuli. Elevated CXCR4 expression has been associated previously with higher CXCR4 signaling (Doijen et al., 2017), indicating that upregulation of CXCR4 on AML cells is responsible for the CXCR4 signaling, potentially related to an increase in CXCR4 homodimer formation in the absence of ligand stimulation (Babcock et al., 2003).

Apart from *Cxcr4*, the screen also identified *Cd47* and *Cd244* (also referred to as 2B4) as positive regulators of *MLL-AF9* leukemia cells *in vivo*; both are known to play a role in immune regulation in normal hematopoiesis and to promote AML cell growth and survival (Majeti et al., 2009; Zhang et al., 2017). *Lrp10* was the only negative regulator of AML cells identified here and has not been associated previously with leukemia. Interestingly, LRP10 is a negative regulator of Wnt/β-catenin signaling (Jeong et al., 2010), a pathway for self-renewal of LSCs (Wang et al., 2010). *Pira6* was also among the top-ranked genes, but because the *Pira6* sgRNAs that were depleted in the screen had homology to several members of the *Pira* family and, therefore, are expected to cause genome instability (Aguirre et al., 2016; Munoz et al., 2016), *Pira6* was a putative false positive hit.

Taken together, we established a CRISPR screen targeting genes encoding cell surface proteins and identified *in vivo* dependencies of *MLL-AF9* leukemia cells. Because the results are limited to a murine *MLL-AF9* leukemia model, it is currently unclear whether they extend to other types of leukemia. Our findings reveal a critical *in vivo* role of CXCR4 signaling in LSCs independent of CXCL12 stimulation. Further, CXCR4 signaling protects *MLL-AF9* AML cells from oxidative stress and differentiation. Our findings suggest that, for significant AML growth inhibition, therapeutic strategies targeting CXCR4 should be aimed at inhibiting CXCR4 signaling rather than blocking the CXCR4-CXCL12 interaction, which restrains AML development.

## STAR★METHODS

### RESOURCE AVAILABILITY

#### Lead Contact

Further information and requests for resources and reagents should be directed to and will be fulfilled by the Lead Contact Dr. Marcus Järås (marcus.jaras@med.lu.se).

#### Materials Availability

All unique/stable reagents generated in this study will be provided by the Lead Contact upon request.

#### Data and Code Availability

The accession number for the RNA sequencing data reported in this paper is Gene Expression Omnibus (GEO): GSE135275.

### EXPERIMENTAL MODEL AND SUBJECT DETAILS

#### Murine leukemia model

The murine AML model was previously generated by retroviral expression of *MLL-AF9* in dsRed C57BL/6 transgenic mice (6051; Jackson Laboratory, Bar Harbor, ME, USA) (Hartwell et al., 2013). The leukemia cells were serially propagated in sublethally irradiated (600 cGy) C57BL/6 recipient mice to minimize irradiation effects and the need for bone marrow support cells. Constitutive lentiviral Cas9 expression was introduced into the dsRed^+^ leukemia cells by transducing pLKO5d.EFS.SpCas9.P2A.PAC into secondary transplanted leukemia cells. Following puromycin (2 μg/mL) selection for three days, the cells were transplanted into sublethally irradiated recipient mice. All experiments were performed with quaternary transplanted leukemia cells. Primitive leukemia cells were enriched by crushing the femurs from leukemic mice followed by red blood cell lysis using NH_4_Cl solution (Stem cell technologies, Vancouver, Canada). All experiments with murine *MLL-AF9* leukemia cells were initiated using c-Kit^+^ cells to enrich for LSCs (Wang et al., 2010). c-Kit^+^ leukemia cells were isolated using CD117 microbeads in MACS® cell separation columns (Miltenyi Biotec, Bergisch Gladbach, Germany). The c-Kit-enriched cells were cultured in serum-free expansion medium (SFEM: Stemspan, StemCell Technologies) supplemented with 1% penicillin/streptomycin. During transduction, the medium was supplemented with murine IL3 (mIL3; 20 ng/mL), murine stem cell factor (mSCF; 50 ng/mL) and human IL6 (hIL6; 20 ng/mL), and spinoculation with the viral supernatant was performed at 650 × *g* for 1 hour. For all transplantation experiments, the mice were sublethally irradiated (600 cGy) prior to injection of leukemia cells. When transplanting transduced leukemia cells, each mouse was injected with cells from separate transductions. Mice of both sexes were included in this study. All the recipient mice were 6–10 weeks old, drug or test naive and were not involved in previous procedures. The mice were gender and age matched and a maximum of 5 mice were used per cage. All experimental mice received autoclaved water and clean rodent chow diet *ad libitum* and were maintained in individually ventilated cages. The animal experiments were conducted according to an Animal Care and Use Committee protocol approved by the Lund/Malmö Ethical Committee.

#### Cxcl12^−/−^ mouse models

*Tek-Cre*^+^ (stock no. 008863), *Prx1-Cre*^+^ (stock no. 005584) and *Ubc-Cre-ERT2*^+^ (*Ubc-Cre*^+^) (stock no. 007001) mice were procured from Jackson Laboratory. *Cxcl12*^*f/f*^ mice (loxP sites flanking exon 2) were crossed with *Tek-Cre*^+^, *Prx1-Cre*^+^ and *Ubc-Cre*^+^ mice to generate *Cxcl12*^*f/f*^-*Tek-Cre*^+^ (*Cxcl12*^−/−^ EC) (Agarwal et al., 2019), *Cxcl12*^*f/f*^-*Prx1-Cre*^+^ (*Cxcl12*^−/−^ MPC) (Agarwal et al., 2019), and *Cxcl12*^*f/f*^-*Ubc-Cre*^+^ (*Cxcl12*^−/−^) mice. Loss of CXCL12 expression in endothelial cells (*Cxcl12*^−/−^ EC) and mesenchymal progenitor cells (*Cxcl12*^−/−^ MPC) from these mice was previously confirmed by real time PCR (Agarwal et al., 2019). Global deletion of *Cxcl12* in *Cxcl12*^*f/f*^-*Ubc-Cre*^+^ mice was achieved by administration of 50 mg/kg of tamoxifen (Sigma-Aldrich; Cat no. T5648) in corn oil through intraperitoneal injections for five consecutive days. All mice were maintained in an AAALAC-accredited animal facility, and all procedures were carried out in accordance with federal guidelines and protocols approved by the Institutional Animal Care and Use Committee at the University of Alabama, Birmingham.

### METHOD DETAILS

#### Global gene expression analysis

To select genes for the screen, we compared the gene expression profiles of *MLL-AF9*-induced murine leukemic GMP with the gene expression profiles of normal mouse GMP and HSC (Affymetrix 430A microarrays; NCBI Gene Expression Omnibus accessions GSE3725 and GSE20377) (Krivtsov et al., 2006; Wang et al., 2010) using Smyth’s moderated t test (Smyth, 2005). To enrich for genes encoding cell surface receptors, we selected 97 genes annotated with the Gene Ontology terms GO0004872 (“Receptor activity”), GO0007155 (“Cell adhesion”), and GO0007166 (“Cell surface signaling”) (http://geneontology.org/) and used manual curation involving literature searches to arrive at a final screening set of 96 genes (Table S1).

#### Generation of viral vectors

pLKO5d.EFS.SpCas9.P2A.PAC (Addgene plasmid #58329), pLKO5.sgRNA.EFS.GFP (Addgene plasmid #57822) and pLKO5.sgRNA.EFS.tRFP657 (Addgene plasmid #57824) were donated by Benjamin Ebert (Heckl et al., 2014). pLKO5.sgRNA.EFS.GFP was linearized using the *Bsmb1* restriction enzyme, and individual sgRNAs targeting *Cxcr4* and *Lrp10* were cloned into the plasmid as described previously (Sanjana et al., 2014). *Cxcr4*^*WT*^, *Cxcr4*^*D99G*^ and *Cxcr4*^*L251P*^ cDNAs were synthesized (Genscript). Silent mutations in the *Cxcr4* sgRNA-binding regions of these cDNAs were included to make them resistant to the specific sgRNAs. The cDNAs were flanked with *Ecor1* and *Xho1* restriction sites for cloning into pMSCV-IRES-GFP (pMIG) as described previously (Peña-Martínez et al., 2018). The lentiviral particles were produced with VSV-G pseudotyping, and gamma-retroviral vectors were produced with eco-tropic pseudotyping in HEK293T cells using standard procedures. The viral supernatants were harvested after 48 hours.

#### Lentiviral CRISPR library generation and sequencing

The lentiviral library was generated essentially as previously described (Sanjana et al., 2014). In brief, 5 sgRNAs were designed for each gene using the sgRNA designer tool (Broad Institute). *Hoxa9* (essential gene) and a non-targeting negative control sgRNA were also included resulting in a library of 486 unique sgRNAs (Table S3). The oligo pool was ordered from CustomArray Inc, which was subsequently hybridized and ligated into the pLKO5.sgRNA.EFS.GFP vector using Gibson assembly (New England Biolabs). Following electroporation of the plasmids into *E. coli*, on average, 120 bacterial colonies for each sgRNA were harvested from agar plates and plasmids isolated using the GeneJET Plasmid Maxiprep Kit (Thermo Fisher). To check the representation of the sgRNAs in the library, we sequenced them in the plasmid pool and in the genomic DNA of *MLL-AF9* leukemia cells transduced with the corresponding pooled viral library. The sgRNAs were PCR amplified using the primer pair 5′ GTCTCGTGGGCTCGGAGATGTGTATAAGAGACAGATATCTTGTGGAAAGGACGAAACAC 3′ and 5′ TCGTCGGCAGCGTCAGATGTGTATAAGAGACAGTTTCAAGTTGATAACGGACTAGCC 3′. The PCR product was purified using Agentcourt AMPure XP beads (Beckman Coulter), and a second PCR was performed to add Nextera XT Index Kit v2 (Illumina) sequencing adapters to the amplicons. The PCR products were again purified using magnetic beads, and the DNA concentrations were determined using the Qubit dsDNA Assay Kit (Invitrogen). The samples were then pooled prior to sequencing in a NextSeq 500 Desktop Sequencer (Illumina) using the NextSeq 500/550 Mid Output v2 Kit, 150 cycles (Illumina), according to the manufacturer’s instructions. The fastq files were aligned using Bowtie 2 to custom reference sequences of the sgRNA library, and the read counts were obtained using Samtools.

#### *In vivo* CRISPR screen

Freshly isolated Cas9^+^c-Kit^+^dsRed^+^ leukemia cells were transduced with the lentiviral CRISPR library by spinoculation as described above. Twenty-four hours post transduction, the cells were transplanted into sublethally irradiated (600 cGy) recipient mice. The mice were sacrificed on day 12, and the bone marrow cells were harvested. Genomic DNA was isolated from the cells collected at 24 hours (T_0_) post transduction and on day 12 (T_12_) post transplantation using a Blood and Cell Culture Kit (QIAGEN). For each sample, a minimum of 4.5 μg (corresponding to on average ~500 cells per sgRNA) of genomic DNA was used for PCR amplification, and the representation of sgRNAs was assessed by next-generation sequencing (NGS) as described above. The raw reads were then normalized in Excel (Microsoft), and the *in vivo* fold-change of the sgRNAs was calculated by dividing the representation of each sgRNA at T_12_ versus T_0._ The sgRNAs were ranked based on the median fold-change of the biological replicates.

#### Flow cytometry and antibody staining

Flow cytometric analyses were performed using a LSRFortessa cell analyzer (Becton Dickinson; BD), and cell sorting was performed using a FACSAria Fusion (BD). For harvesting of leukemia cells from mice, femurs and tibias were crushed and spleens were mashed, followed by filtration using a 70-μm cell strainer. Red blood cell lysis was performed using NH_4_Cl solution. Anti-mouse CXCR4 antibodies conjugated to APC (REA107, Miltenyi Biotec) and BV711 (L276F12, Biolegend) were used. Anti-mouse Gr-1-APC-Cy7 (RB6–8C5), anti-mouse CD117-APC (2B8), anti-mouse CD117-AF700 (2B8) antibodies and Annexin V-APC were obtained from Biolegend. A ROS assay kit 520nm (Thermo Fischer Scientific) and CellROX Deep Red reagent (Thermo Fischer Scientific) were used to measure intracellular ROS levels. Flow cytometric data were analyzed using FlowJo (FlowJo LLC). For phospho-flow analysis, the cells were fixed using paraformaldehyde (1.6%) for 10 min at room temperature followed by permeabilization using paraformaldehyde ice-cold ethanol (99%) and immediately vortexed and washed with phosphate buffered saline. The cells were stained with the antibodies specific for phosphorylated forms of the intracellular protein NF-κB-PE/Cy7, p38 MAPK-PE/Cy7 and ERK-BV421 (20A) (BD Biosciences).

#### Quantification of CRISPR editing

Cas9^+^dsRed^+^ *MLL-AF9* leukemia cells were transduced with lentiviral *Cxcr4* sgRNA vectors coexpressing GFP and cultured for 3 days. Sorting of GFP^+^ cells was performed by flow cytometry followed by genomic DNA isolation. The binding regions of *Cxcr4* sgRNA_a and *Cxcr4* sgRNA_b were PCR amplified using the primer pairs 5′TCCACAGGCTATCGGGGTAA3′, 5′GTGACGTTGTCTGTCCCTGT3′ and 5′ATCTGTGACCGCCTTTACCC3′, 5′TCCTGCCTAGACACTCATTCAC3′, followed by amplicon tagmentation prior to NGS (Illumina). CRISPR-mediated DNA editing was quantified using the bioinformatics tool TIGERq (TIGERq, Lund, Sweden).

#### Bone marrow homing assay

Cas9^+^c-Kit^+^dsRED^+^ leukemia cells were transduced with sgRNA-expressing lentiviral vectors coexpressing GFP and cultured for 3 days. The percentage of GFP^+^ cells was assessed by flow cytometry, and then 3×10^6^ cells were transplanted into sublethally irradiated mice. Femurs of the recipient mice were harvested after 24 hours, and the percentages of GFP^+^ cells within leukemic (dsRed^+^) cells were determined by flow cytometric analysis. Similarly, in the homing experiment with sorted cells, 600,000 GFP^+^ cells were transplanted into sublethally irradiated mice and the percentage of GFP^+^ cells in the bone marrow was assessed 24 hours later.

#### *In vivo* rescue assays with mutated Cxcr4 variants

Cas9^+^c-Kit^+^dsRed^+^ leukemia cells were transduced with the *Cxcr4* sgRNA_b lentiviral vector coexpressing tRFP657. After 24 hours, the cells were washed and transduced with retroviral vectors expressing *Cxcr4*^*WT*^, *Cxcr4*^*D99G*^ or *Cxcr4*^*L251P*^. An empty vector without a *Cxcr4* insert was used as a control. Twenty-four hours after the second transduction, the cells were washed and plated in serum-free medium supplemented with standard cytokines (see above). Forty-eight hours after the second transduction, the cells were washed, and a fraction of the cells was analyzed by flow cytometry to determine the input percentage of GFP^+^ cells within dsRed^+^tRFP657^+^ cells. The remaining cells were transplanted into sublethally irradiated (600 cGy) C57BL/6 recipient mice. The mice were sacrificed 14 days later, and their bone marrow and spleen were analyzed to quantify the percentage of GFP^+^ cells within the dsRed^+^tRFP657^+^ cell population.

#### Real-time PCR analysis

RNA was extracted from the tail tissue of *Cxcl12*^*f/f*^-*Ubc-Cre*^+^ and *Cxcl12*^*f/f*^*-Ubc-Cre*^−^ mice using an RNeasy micro kit (QIAGEN, Valencia, CA), and cDNA was synthesized using the Superscript III First-Strand Kit (Invitrogen, Grand Island, NY). Quantitative real-time PCR was performed using a TaqMan probe for mouse CXCL12 (Mm00445553_m1) and GAPDH (4352932E) as an endogenous control in a QuantStudio 6 Flex Real-Time PCR System.

#### Cell cycle analysis

dsRed^+^ leukemia cells harvested from *Cxcl12*^*f/f*^-*Ubc-Cre*^+^ and *Cxcl12*^*f/f*^*-Ubc-Cre*^−^ mice were fixed and permeabilized using BD Cytofix/Cytoperm (BD). Then, the cells were stained with anti-Ki-67 (eBiosciences) and DAPI prior to analyzing their cycle status by flow cytometry.

#### Culture conditions with CXCR4 ligands

c-Kit^+^dsRed^+^ leukemia cells were cultured in SFEM (StemCell Technologies) with 1% penicillin/streptomycin supplemented with increasing concentrations of CXCL12 (CHM324, Prospec), recombinant MIF (300–69, Peprotech), UBIQUITIN (U6253–5MG, Sigma) or without any cytokine in a 96-well plate. Cells were counted using count bright beads (Thermo Fisher) using flow cytometry.

#### RNA sequencing

Ten days post transplantation of dsRed^+^ leukemia cells transduced with control or *Cxcr4* sgRNA-expressing lentiviral vectors, leukemia cells were harvested from the mice. GFP^+^ (coexpressed with sgRNA) was sorted by FACS, and RNA was extracted using a RNeasy kit (QIAGEN). RNA sequencing libraries were prepared using the TruSeq RNA Sample prep kit v2 (Illumina, San Diego, CA, USA), and sequencing was performed using the NextSeq 500/550 Mid Output v2 Kit, 150 cycle (Illumina). The sequenced reads were aligned to mm9 mouse reference genomes using TopHat 2.0.13 (Kim et al., 2013). Differential gene expression analysis and visualization of the transcript data were performed using Qlucore omics Explorer 3.0 (Qlucore, Lund, Sweden). Gene expression levels were compared between the control and *Cxcr4* sgRNA groups by performing t tests. The logarithms of the *p* values and the signs of the fold-change were used to prepare ranked gene lists that were used for gene set enrichment analysis (GSEA) (Subramanian et al., 2005). HALLMARK (H), CURATED (C2) and GENE ONTOLOGY (C5) gene sets were downloaded from Molecular Signatures Database (MSigDB) collections.

#### Cytology

sgRNA-expressing leukemia cells were harvested from the bone marrow of recipient mice 13 days post transplantation. GFP^+^ cells (coexpressed with sgRNA) were sorted by FACS and were subjected to cytospin preparation onto glass slides. The samples were stained with May-Grünwald (Merck) and Giemsa (Merck) for microscopic imaging using Nikon Eclipse 50i microscope at 100x magnification with oil immersion. The images were acquired using Leica DFC320 camera and Leica IM500 acquisition software.

### QUANTIFICATION AND STATISTICAL ANALYSIS

Prism 6 (GraphPad) was used for the statistical analyses, including Student’s t test, linear regression analysis and Kaplan-Meier survival analysis. Significance is depicted with asterisks: *p < 0.05, **p < 0.01, ***p < 0.001, ****p < 0.0001. Data are presented as the mean ± standard deviation. Information about the number of biological replicates (n) and the *p* values are provided in the figure legends.

## Supplementary Material

Supplementary Table 3

1

## Figures and Tables

**KEY RESOURCES TABLE:** 

REAGENT or RESOURCE	SOURCE	IDENTIFIER
Antibodies		
Anti mouse CD184 (CXCR4) – APC	Miltenyi Biotec	Cat# 130-102-245; RRID: AB_2655759
Anti mouse CD184 (CXCR4) – BV711 (Clone L276F12)	Biolegend	Cat# 146505; RRID: AB_2562782
Anti mouse GR1 – APC-Cy7 (Clone RB6–8C5)	Biolegend	Cat# 108423; RRID: AB_2137486
Anti mouse phosphorylated p38 – PE-Cy7 (pT180/pY182) (Clone 36/p38)	BD Biosciences	Cat# 560241; RRID: AB_1645297
Anti mouse phosphorylated NF-κB – PE-Cy7 (pS529) (Clone K10–895.12.50)	BD Biosciences	Cat# 560335; RRID: AB_1645545
Anti mouse ERK1/2 – BV421 (pT202/pY204) (Clone 20A)	BD Biosciences	Cat# 561991; RRID: AB_10895978
Anti mouse CD117 microbeads antibody	Miltenyi Biotec	Cat# 130–091-224; RRID: AB_2753213
Anti mouse CD117 – APC (Clone 2B8)	Biolegend	Cat# 105812; RRID: AB_313221
Anti mouse CD117 – AF700 (Clone 2B8)	Biolegend	Cat# 105845; RRID: AB_2783045
Anti-Ki67	Thermo Fisher Scientific	Cat#11–5698-80; RRID: AB_11151689
Annexin V - APC	Biolegend	Cat# 640920

Bacterial and Virus Strains		

pLKO5d.EFS.SpCas9.P2A.PAC	Heckl et al., 2014	RRID: Addgene_58329
pLKO5.sgRNA.EFS.GFP	Heckl et al., 2014	RRID: Addgene_57822
pLKO5.sgRNA.EFS.tRFP657	Heckl et al., 2014	RRID: Addgene_57824

Chemicals, Peptides, and Recombinant Proteins		

Mouse CXCL12	Prospec Bio	Cat# CHM-324
Recombinant human MIF	Peprotech	Cat# 300–69
Ubiquitin from bovine erythrocytes	Sigma	Cat# U6253
Recombinant human IL-6	Peprotech	Cat# 200–06
Recombinant mouse SCF	Peprotech	Cat# 250–03
Recombinant mouse IL-3	Peprotech	Cat# 213–13
Ammonium Chloride (NH_4_Cl)	Stem Cell Technologies	Cat# 07800
N-Acetyl L-Cysteine	Sigma Aldrich	Cat# A8199–10G

Critical Commercial Assays		

RNeasy Micro Kit	QIAGEN	Cat# 74004
Nextera XT DNA Library Preparation Kit	Illumina	Cat# FC-131–1096
Blood and Cell Culture Kit	QIAGEN	Cat# 13343
GeneJET Plasmid Maxiprep Kit	Thermo Fischer Scientific	Cat# K0491
NextSeq 500/550 v2 mid output kit (upgraded to v2.5)	Illumina	Cat# 20024907
TruSeq RNA Library Preparation Kit v2	Illumina	Cat# RS-122–2001
Agencourt AMPure XP	Beckman Coulter	Cat# A63880
Total Reactive Oxygen Species (ROS) Assay Kit 520 nm	Thermo Fischer Scientific	Cat# 88–5930-74; RRID: AB_2574932
CellROX Deep Red Reagent	Thermo Fischer Scientific	Cat# C10422

Deposited Data		

RNA sequencing data	This paper	GEO: GSE135275

Experimental Models: Organisms/Strains		

Mouse: C57BL/6JRj	Janvier Labs	N/A
Mouse: B6.Cg-Tg(Tek-cre)1Ywa/J	Jackson Laboratory	Stock No: 008863
Mouse: B6.Cg-Tg(Prrx1-cre)1Cjt/J	Jackson Laboratory	Stock No: 005584
Mouse: B6.Cg-Ndor1-Tg(UBC-cre/ERT2)1Ejb/1J	Jackson Laboratory	Stock No: 007001

Oligonucleotides		

mCxcl12 qPCR probe	Thermo Fisher Scientific	Cat# Mm00445553_m1
mGAPDH qPCR probe	Thermo Fisher Scientific	Cat# 4352932E

Recombinant DNA		

pMIG-CXCR4^WT^	This paper	N/A
pMIG-CXCR4^D99G^	This paper	N/A
pMIG-CXCR4^L251P^	This paper	N/A

Software and Algorithms		

FlowJo software (version 8.5.2)	FlowJo	RRID:SCR_008520
BD FACSDiva	BD Biosciences	RRID:SCR_001456
GraphPad Prism 7	GraphPad	RRID:SCR_002798
Qlucore omics Explorer 3.0	Qlucore	N/A
Bowtie2	Langmead and Salzberg, 2012	http://bowtie-bio.sourceforge.net/bowtie2/index.shtml
Samtools	Li et al., 2009	http://samtools.sourceforge.net/
TopHat 2.0.13	Kim et al., 2013	https://github.com/infphilo/tophat

## Highlights

- *In vivo* CRISPR screening identifies CXCR4 as a key regulator of AML stem cells
- CXCL12 expression in the bone marrow is dispensable for AML development
- CXCR4 signaling protects AML cells from oxidative stress and differentiation
