# Supplementary material for: CXCR4 Signaling Has a CXCL12-Independent Essential Role in Murine MLL-AF9-Driven Acute Myeloid Leukemia
Source: Cell Rep. Author manuscript; Available in PMC 2021 May 10. (PMC8109054; doi:10.1016/j.celrep.2020.107684)
Supplement: 1 [file NIHMS1693424-supplement-1.pdf]

Cell Reports, Volume *31*

Supplemental Information

CXCR4 Signaling Has a CXCL12-Independent Essential Role in Murine *MLL-AF9*-Driven Acute Myeloid Leukemia

Ramprasad Ramakrishnan, Pablo Peña-Martínez, Puneet Agarwal, Maria Rodriguez-Zabala, Marion Chapellier, Carl Högberg, Mia Eriksson, David Yudovich, Mansi Shah, Mats Ehinger, Björn Nilsson, Jonas Larsson, Anna Hagström-Andersson, Benjamin L. Ebert, Ravi Bhatia, and Marcus Järås

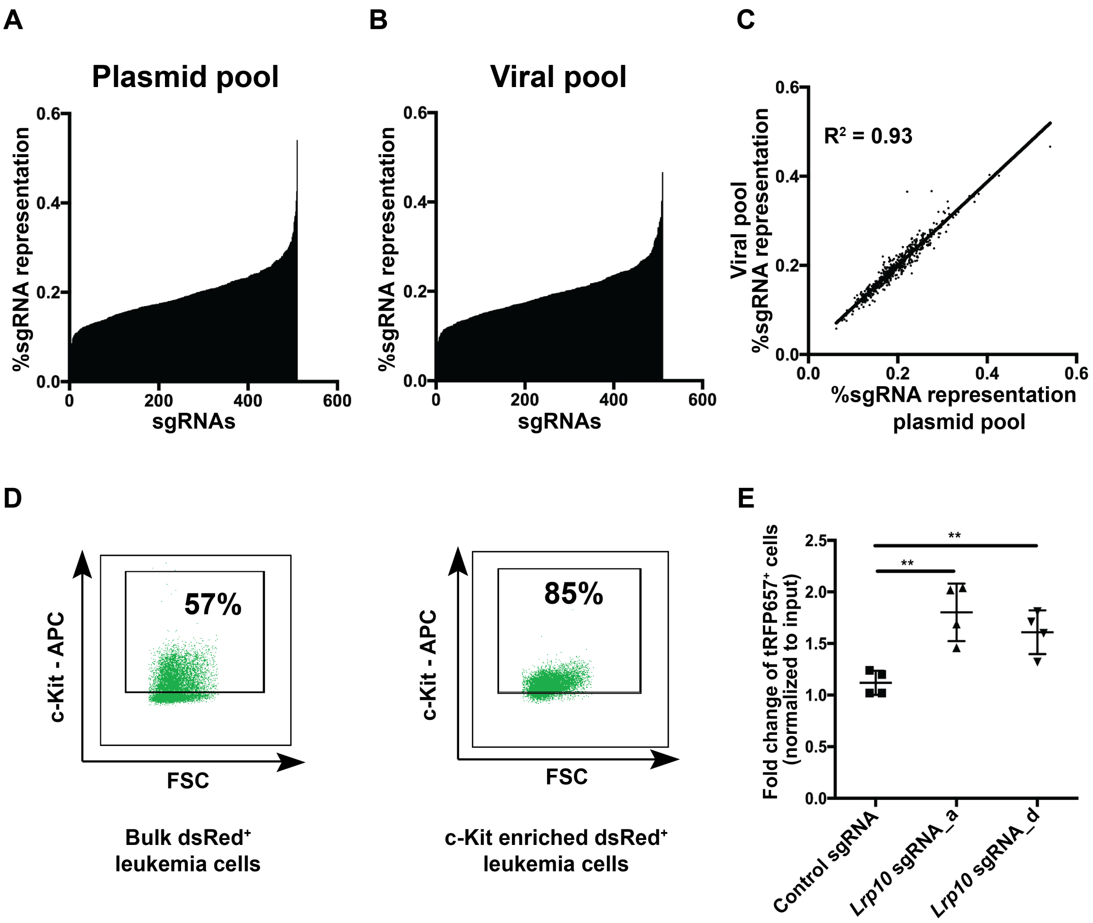

Figure S1. **sgRNA representation, enrichment of c-Kit$^+$ leukemia cells, and CRISPR-mediated disruption of *Lrp10* accelerates leukemia development *in vivo* (Related to Figure 1).** Representation of sgRNAs determined by next-generation sequencing in the (**A**) plasmid and (**B**) viral pool as assessed post transduction in *MLL-AF9* leukemia cells. (**C**) Linear regression analysis of the representation of sgRNAs in the plasmid and the viral pool. (**D**) Dot plots showing c-Kit expression in *MLL-AF9* dsRed$^+$ mononuclear leukemia cells before (bulk cells) and after c-Kit enrichment. (**E**) Mice (n=4 for each group) were transplanted with leukemia cells transduced with lentiviral vectors coexpressing *Lrp10* sgRNAs and tRFP657. The mice were sacrificed 16 days post transplantation, and the percentage of tRFP657$^+$ cells in the bone marrow was normalized to the input percentage of tRFP657$^+$ cells 2 days after transduction. Means and standard deviations are shown (**, $P < 0.01$).

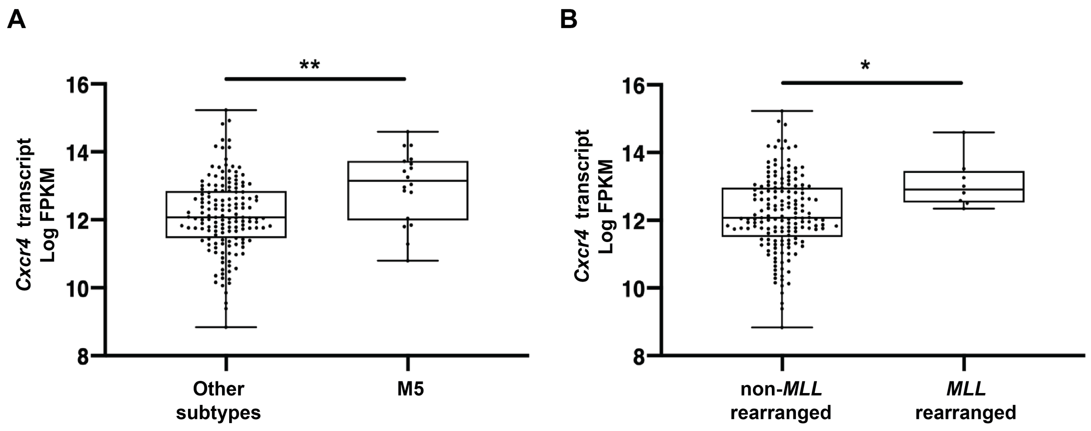

Figure S2. ***Cxcr4* expression is elevated in M5 and *MLL*-rearranged AML** (**Related to Figure 2**). *Cxcr4* expression in (**A**) M5 compared to other subtypes of AML and (**B**) *MLL*-rearranged AML compared to non-*MLL*-rearranged AML in patient samples from the cancer genome atlas (TCGA). Four patient samples in the M5 AML subtype contained *MLL*-rearrangements. FPKM: Fragments per Kilobase of transcript per Million mapped reads; Box represents 25th to 75th percentile of the data. Medians and minimum to maximum are shown (*, P<0.05; **, P<0.01).

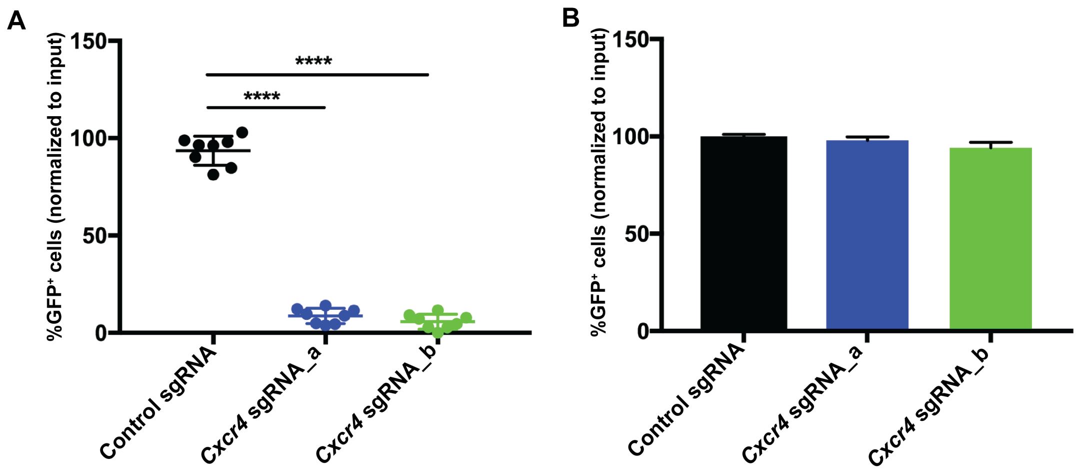

Figure S3. **CRISPR-mediated disruption of *Cxcr4* depletes leukemia cells *in vivo* but not *in vitro* (Related to Figure 2).** (**A**) Mice (n=8 for each group) were transplanted with leukemia cells transduced with lentiviral vectors coexpressing *Cxcr4* sgRNAs and GFP. The mice were sacrificed either 12 or 13 days post transplantation, and the percentage of GFP^+ cells in the spleen was normalized to the input percentage of GFP^+ cells 2 days after transduction. (**B**) The leukemia cells were transduced with lentiviral vectors coexpressing *Cxcr4* sgRNAs and GFP and the transduction efficiency was determined after 3 days (input). The cells were cultured *in vitro* with IL3, IL6, and SCF for 3 more days (n=3) and the percentage of GFP^+ cells was normalized to the input. Means and standard deviations are shown (****, $P < 0.0001$).

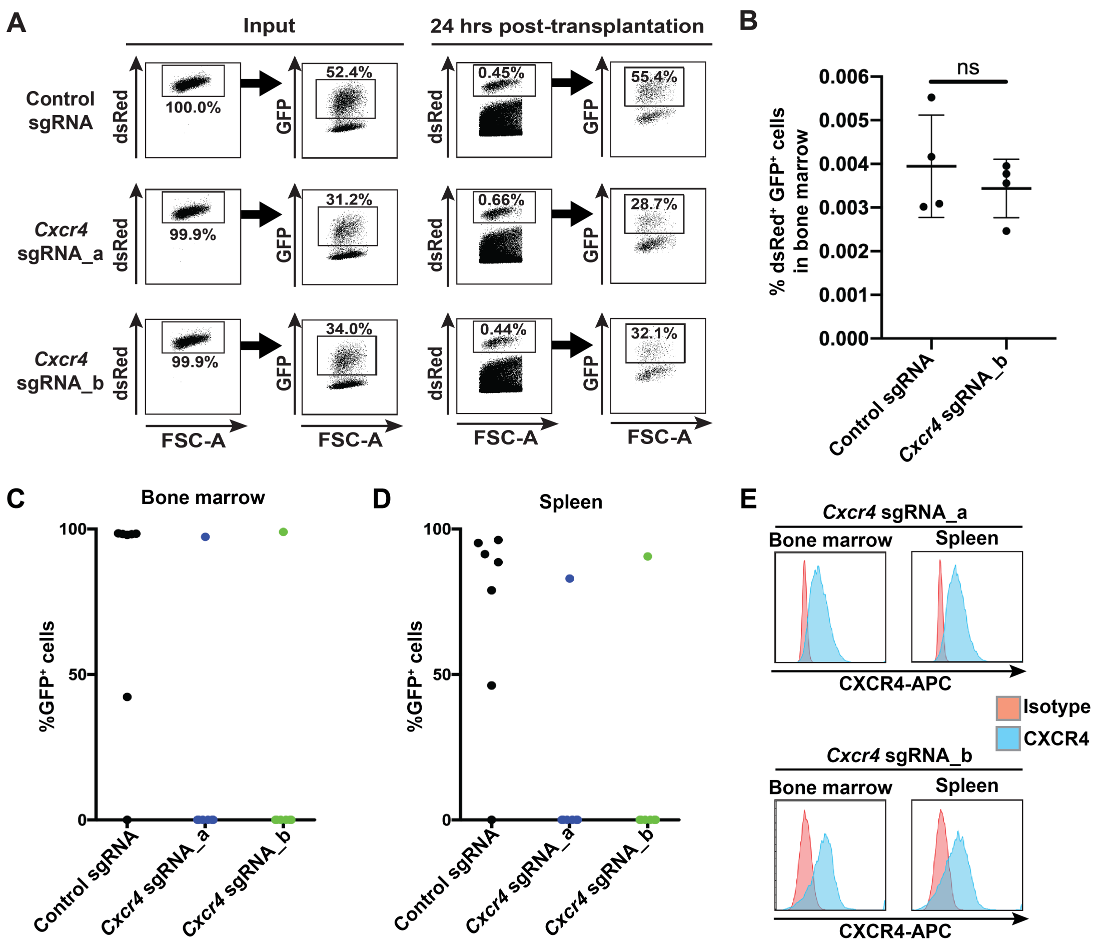

Figure S4. CXCR4 is dispensable for homing of the leukemia cells to the bone marrow but critical for leukemia development (Related to Figure 3). (**A**) Representative FACS plots showing the percentage of unsorted GFP$^+$ cells within dsRed$^+$ leukemia cells prior to transplantation (input) and in the bone marrow 24 hours after transplantation. (**B**) Percentage of dsRed$^+$GFP$^+$ *MLL-AF9* leukemia cells in the bone marrow of recipient mice 24 hours after transplantation (n=4 for each group) of sorted dsRed$^+$GFP$^+$ leukemia cells. Leukemia burden in the bone marrow (**C**) and spleen (**D**) of mice (n=7 for each group) transplanted with sorted GFP$^+$dsRed$^+$ leukemia cells following transduction with sgRNA-expressing lentiviral vectors. (**E**) CXCR4 expression on GFP$^+$dsRed$^+$ leukemia cells from the two mice that had developed leukemia after transplantation of *Cxcr4* sgRNA-expressing leukemia cells. Means and standard deviations are shown (ns: not significant).

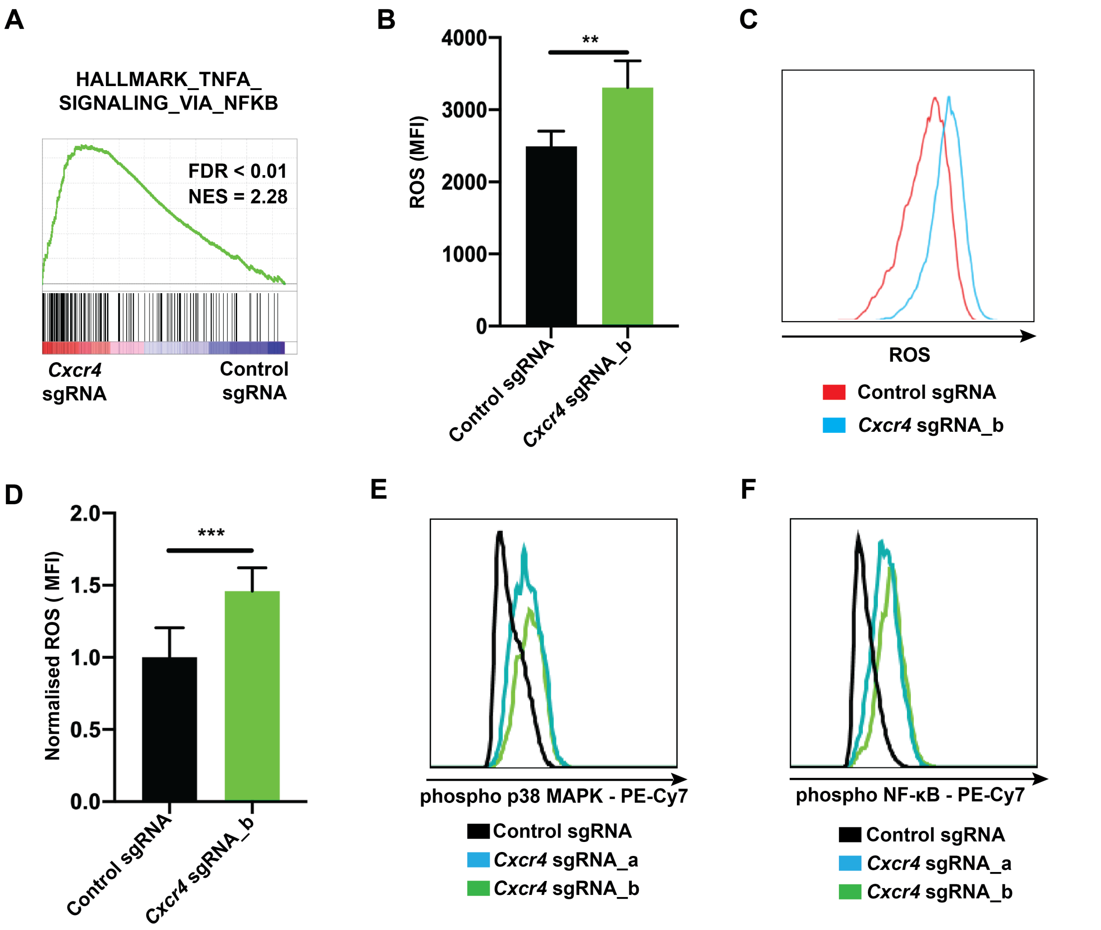

Figure S5. Loss of CXCR4 signaling results in activation of NF-κB and p38 MAPK (Related to Figure 4 and 5). (**A**) Gene set enrichment analysis (GSEA) of NF-κB target genes in the transcriptional signature of leukemia cells with *Cxcr4* disruption. (**B**) Mean fluorescence intensity (MFI) of CellROX Deep Red (measuring total ROS) in c-Kit$^+$ cells within GFP$^+$ (sgRNA expressing) leukemia cells. sgRNA-expressing leukemia cells were harvested from the bone marrow of recipient mice (n=5 for control sgRNA; n=4 for *Cxcr4* sgRNA_b) 14 days post transplantation. (**C**) Representative histogram showing total ROS levels (measured with H2DCFDA) within tRFP657$^+$ (sgRNA expressing) cells harvested from the bone marrow of recipient mice (n=7 for each group) 11 days post transplantation. (**D**) MFI of H2DCFDA (measuring total ROS) within tRFP657$^+$ (sgRNA expressing) cells harvested from the bone marrow of recipient mice (n=7 for each group) 11 days post transplantation. The data is pooled from two independent experiments and is normalized to the average H2DCFDA staining in the control sgRNA samples within each experiment. Representative histogram showing the expression of (**E**) phosphorylated p38 MAPK and (**F**) phosphorylated NF-κB within GFP$^+$ (sgRNA expressing) cells in the bone marrow of mice transplanted with *Cxcr4* disrupted leukemia cells.

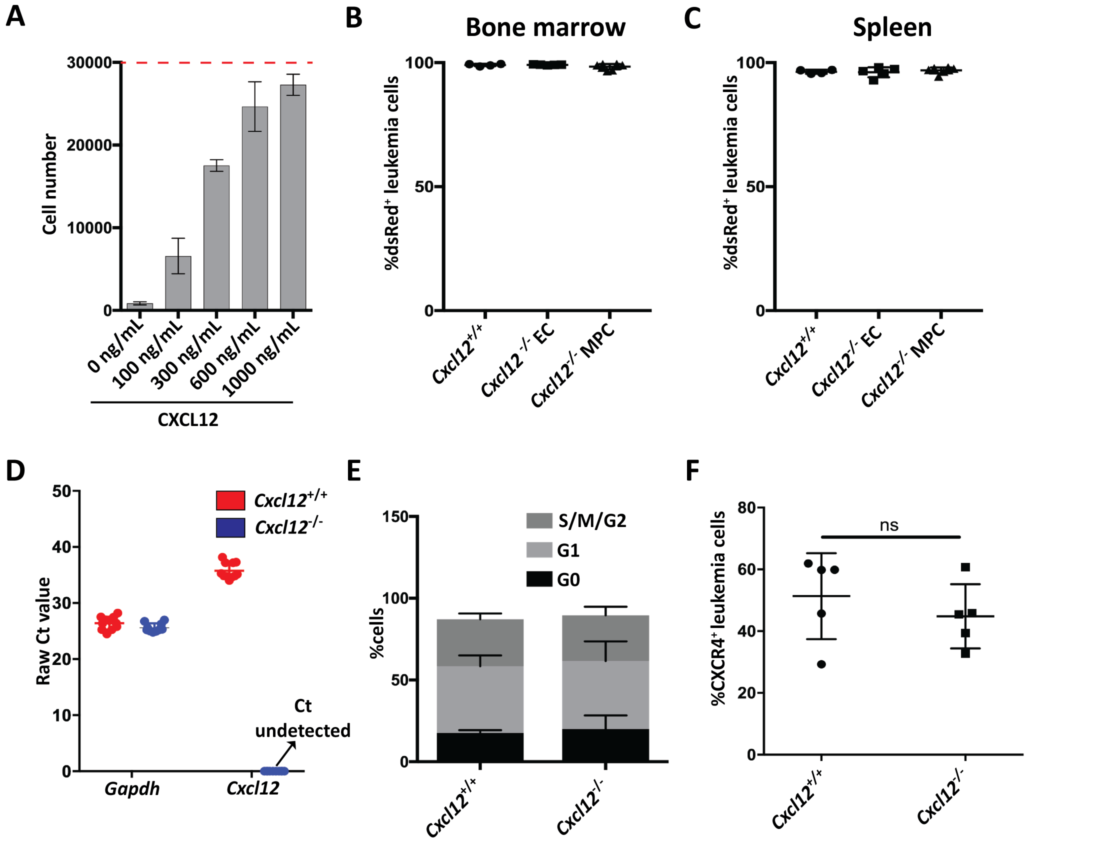

Figure S6. CXCL12 stimulation, leukemia burden, real-time PCR, cell cycle analysis and CXCR4 expression (Related to Figure 6). (A) Cell number after three days of culture of 30,000 seeded dsRed$^+$ leukemia cells (red dotted line) stimulated with increasing concentrations of CXCL12. Percentage of leukemia (dsRed$^+$) cells in *Cxcl12*$^{+/+}$ (n=4), *Cxcl12*$^{-/-}$ EC (n=5) and *Cxcl12*$^{-/-}$ MPC (n=8) recipient mice at the time of sacrifice in the (**B**) bone marrow and (**C**) spleen. (**D**) Real-time PCR analysis (raw Ct values) of *Gapdh* and *Cxcl12* from RNA of the tail tissue of *Cxcl12*$^{f/f}$*-Ubc-Cre*$^+$ (*Cxcl12*$^{-/-}$) and *Cxcl12*$^{+/+}$ mice. (**E**) Cell cycle status and (**F**) CXCR4 expression in the leukemia cells from the bone marrow of *Cxcl12*$^{f/f}$*-Ubc-Cre*$^+$ (*Cxcl12*$^{-/-}$) and *Cxcl12*$^{+/+}$ mice (n=5). Means and standard deviations are shown. ns: not significant, Ct: Cycle threshold, EC: Endothelial cells, MPC: Mesenchymal progenitor cells.

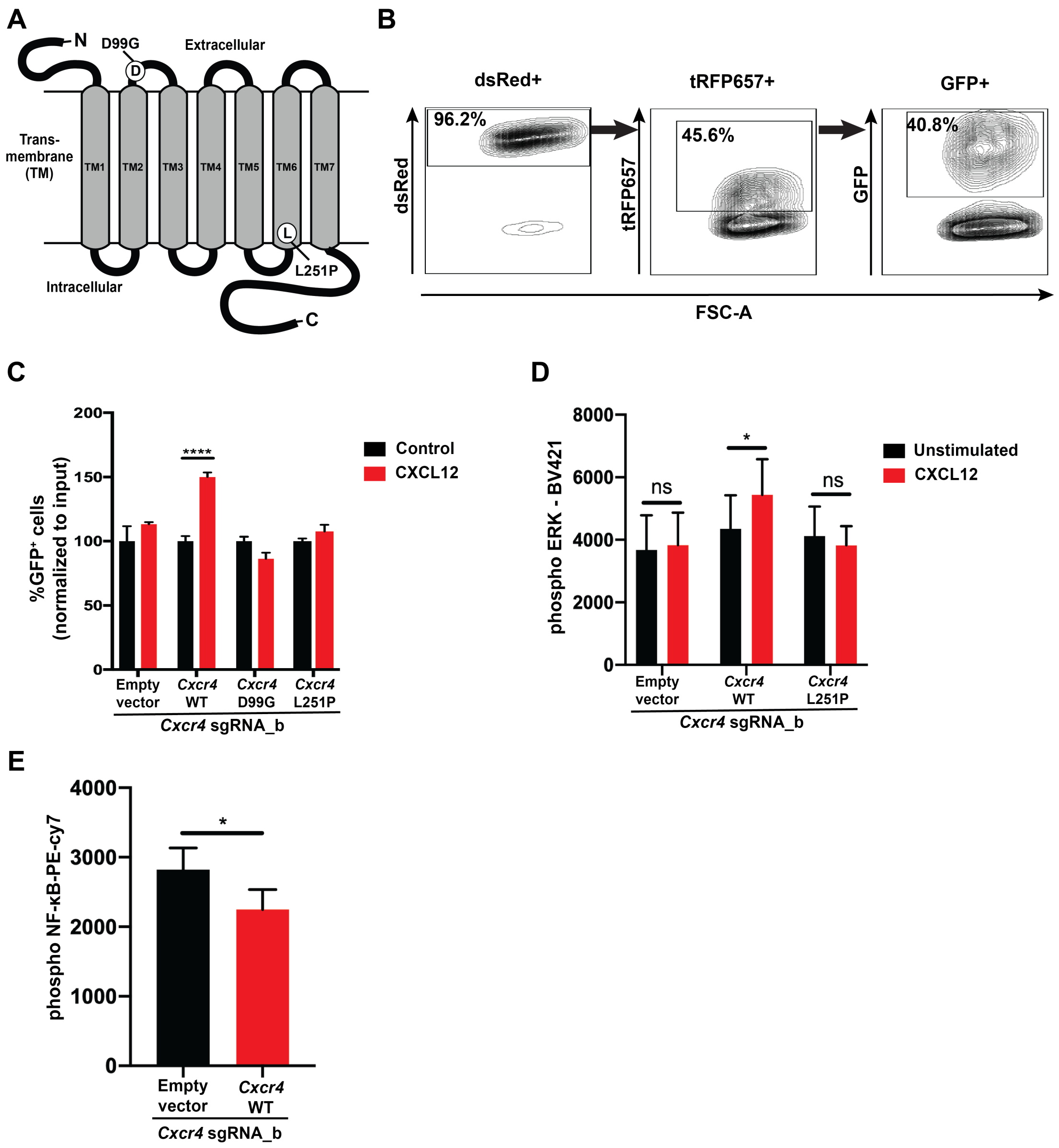

Figure S7. CXCR4 mutants are insensitive to CXCL12 stimulation, and overexpression of *Cxcr4*^{WT} suppresses NF-κB activation (Related to Figure 7). (A) Schematic representation of CXCR4 showing the substitutions of amino acid residues D99G and L251P. **(B)** Representative FACS plots showing the sequential gating strategy for measuring the percentage of GFP$^+$ cells within the dsRed$^+$tRFP657$^+$ leukemia cells. (**C**) Percentage of GFP$^+$ cells within the dsRed$^+$tRFP657$^+$ leukemia cells normalized to the input (3 days post transduction). Cells were cultured for 3 days in serum free medium without any cytokines (control) or with 1 µg/mL of CXCL12. (**D**) Expression of phosphorylated ERK shown as mean fluorescence intensity (MFI) in the leukemia cells co-transduced with *Cxcr4* sgRNA_b and Empty vector, *Cxcr4*^{WT} or *Cxcr4*^{L251P}. dsRed$^+$tRFP657$^+$GFP$^+$ cells were sorted by flow cytometry, then cultured for 3 days and subsequently stimulated with 1 µg/mL of CXCL12 for 2 minutes prior to ERK-staining. Expression of phosphorylated NF-κB (**E**) shown as MFI within FACS sorted GFP$^+$dsRed$^+$tRFP657$^+$ leukemia cells harvested from the bone marrow of mice 13 days post transplantation (n=4 for each group). The recipient mice were transplanted with dsRed$^+$ leukemia cells transduced with *Cxcr4* sgRNA_b coexpressing tRFP657 along with pMIG-Empty vector or *Cxcr4*^{WT} coexpressing GFP. Means and standard deviations are shown (****, $P < 0.0001$; *, $P < 0.05$; ns: not significant).

Supplementary table 1. Ranked genes based on upregulation in leukemic granulocyte-monocyte progenitors (L-GMPs) compared to normal GMPs and hematopoietic stem cells (HSCs). (Related to Figure 1)

Rank	Gene symbol	L-GMP vs GMP (*t*)	L-GMP vs HSC (*t*)
1	*Csf2ra*	15.5	20.4
2	*Ncam1*	14	16.7
3	*Gpr97*	12.1	5.6
4	*Itgb5*	10.8	6.5
5	*P2rx1*	10	5.8
6	*Itgb2*	9.7	9.8
7	*Fcer1g*	7.8	11.8
8	*Emr1*	7.4	8.8
9	*Il31ra*	7.3	11.1
10	*Ifngr1*	7.1	10.9
11	*Cd33*	6.8	6.5
12	*Csf2rb2*	6.6	5.7
13	*Tlr1*	6.5	7.8
14	*Paqr7*	6.4	3.3
15	*Adipor1*	6.3	11.5
16	*Cadm1*	6.2	4.8
17	*Plxnd1*	6.2	8
18	*Lrp4*	5.9	9.1
19	*Cd97*	5.9	6.2
20	*Crlf2*	5.8	0.9
21	*Cd44*	5.7	2.8
22	*4632428N05Rik*	5.6	7.5
23	*Cd79b*	5.4	4.7
24	*Olr1*	5.4	8.6
25	*App*	5.2	11.6
26	*Ccr1*	5.1	6
27	*Il10rb*	5	4.4
28	*Gpr172b*	5	2.9
29	*Aplp2*	5	0.8
30	*Sort1*	4.9	8
31	*Clec4b1*	4.8	6.2
32	*Lmbr1*	4.8	1.5
33	*L1cam*	4.5	4.2
34	*Itgae*	4.4	5.2
35	*Pxn*	4.4	2.5
36	*Tnfrsf13b*	4.4	3.7

Supplementary table 1 (Continued)

Rank	Gene symbol	L-GMP vs GMP (*t*)	L-GMP vs HSC (*t*)
37	*Cd47*	4.4	2.1
38	*Cml2*	4.4	2.8
39	*Vcam1*	4.4	6.6
40	*AF251705*	4.3	4.9
41	*Psen1*	4.3	7.4
42	*Entpd1*	4.2	2.2
43	*Lrpap1*	3.9	1
44	*Erbb3*	3.8	5.6
45	*Sirpa*	3.7	5.8
46	*Cd244*	3.7	1.4
47	*Cxcr4*	3.7	5.6
48	*Gpr19*	3.5	1.8
49	*Sorl1*	3.5	5.4
50	*Pira6*	3.4	3
51	*Rtp3*	3.4	5.4
52	*Ceacam1*	3.4	4.4
53	*Siglec5*	3.3	5.5
54	*Lrp1*	3.3	3.2
55	*Pilra*	3.2	3.6
56	*Thbd*	3.2	4.8
57	*Plp1*	3.2	3.5
58	*Lrp10*	3.2	3.2
59	*Tspo*	3.2	9
60	*Flot2*	3.2	6.5
61	*Ltbr*	3.2	4.4
62	*Itgav*	3.1	2.7
63	*Ltb4r1*	3.1	5.4
64	*Prlr*	3.1	2.7
65	*Cd180*	3.1	3.5
66	*Tnfrsf1a*	2.9	5.4
67	*Itgam*	2.9	3.1
68	*Ptger1*	2.9	3.5
69	*Tacr2*	2.8	1.2
70	*Stab1*	2.8	2.7
71	*Gria3*	2.8	7.6
72	*Fcgr2b*	2.8	9.4
73	*Ptpro*	2.7	3.6
74	*Cnr2*	2.7	4.5

Supplementary table 1 (Continued)

Rank	Gene symbol	L-GMP vs GMP (*t*)	L-GMP vs HSC (*t*)
75	*Clec2i*	2.6	2.7
76	*Ifnar2*	2.6	2.1
77	*Apob48r*	2.6	1.1
78	*Sema4d*	2.6	0.2
79	*Cdh20*	2.5	2.6
80	*Sema4a*	2.4	3.5
81	*Cd80*	2.4	1.4
82	*Gp1bb*	2.4	2.6
83	*Tgfbr1*	2.4	1.5
84	*Ms4a6b*	2.3	3.2
85	*Pcdh10*	2.3	1.8
86	*Epha1*	2.3	4.0
87	*Icam2*	2.2	4.1
88	*Plaur*	2.2	1.4
89	*Gpr84*	2.1	2.6
90	*Ddr1*	2.1	1.5
91	*Msr1*	2.1	1.9
92	*Epha7*	2.1	2.4
93	*Cd300lf*	2.1	2.2
94	*Reep5*	2.1	7.6
95	*Il3ra*	2.0	3.9
96	*Itgal*	1.9	3.1

t : moderated *t* statistics from Smyth's moderated *t*-test

Supplementary table 2. Top ranked positive and negative regulators of leukemia cells in the CRISPR screen. (Related to Figure 1)

Positive regulators of AML cells- ranked list			
Rank	Gene	Number of sgRNAs depleted	Depletion (median fold-change)
1	*Cxcr4*	5/5	-9.73
2	*Hoxa9*	5/5	-4.69
3	*Pira6*	4/5	-4.71
4	*Ifngr1*	4/5	-2.53
5	*CD47*	3/5	-5.84
6	*CD244*	2/5	-3.37
7	*Af251705*	2/5	-3.24
8	*Clec4b1*	2/5	-3.18
9	*Ceacam1*	2/5	-2.82
10	*C10orf57*	2/5	-2.31
Negative regulator of AML cells			
Rank	Gene	Number of sgRNAs enriched	Enrichment (median fold-change)
1	*Lrp10*	4/5	2.77

Threshold for sgRNA depletion or enrichment: fold-change of +/- 2.0

**Ranking criteria**	
1	Number of sgRNAs depleted or enriched per gene
2	The median fold-change in representation of sgRNAs per gene.
